# Differential cell adhesion implemented by *Drosophila* Toll corrects local distortions of the anterior-posterior compartment boundary

**DOI:** 10.1038/s41467-020-20118-y

**Published:** 2020-12-10

**Authors:** Norihiro Iijima, Katsuhiko Sato, Erina Kuranaga, Daiki Umetsu

**Affiliations:** 1 Laboratory for Histogenetic Dynamics, Graduate School of Life Sciences, Tohoku University, Sendai, 980-8578 Japan; 2 Research Institute for Electronic Science, Hokkaido University, Sapporo, 001-0020 Japan; 3 Global Station for Soft Matter, Global Institution for Collaborative Research and Education, Hokkaido University, Sapporo, 001-0020 Japan

**Keywords:** Cell adhesion, Morphogenesis

## Abstract

Maintaining lineage restriction boundaries in proliferating tissues is vital to animal development. A long-standing thermodynamics theory, the differential adhesion hypothesis, attributes cell sorting phenomena to differentially expressed adhesion molecules. However, the contribution of the differential adhesion system during tissue morphogenesis has been unsubstantiated despite substantial theoretical support. Here, we report that Toll-1, a transmembrane receptor protein, acts as a differentially expressed adhesion molecule that straightens the fluctuating anteroposterior compartment boundary in the abdominal epidermal epithelium of the *Drosophila* pupa. *Toll-1* is expressed across the entire posterior compartment under the control of the selector gene *engrailed* and displays a sharp expression boundary that coincides with the compartment boundary. *Toll-1* corrects local distortions of the boundary in the absence of cable-like Myosin II enrichment along the boundary. The reinforced adhesion of homotypic cell contacts, together with pulsed cell contraction, achieves a biased vertex sliding action by resisting the separation of homotypic cell contacts in boundary cells. This work reveals a self-organizing system that integrates a differential adhesion system with pulsed contraction of cells to maintain lineage restriction boundaries.

## Abstract (web summary)

The differential adhesion hypothesis is proposed to play a role during epithelial tissue morphogenesis but it has remained unclear. Here, the authors identify the Toll-1 receptor as a differentially expressed adhesion molecule that maintains lineage restriction boundaries in the *Drosophila* epidermal epithelium.

## Introduction

Vertebrate and invertebrate tissues are often subdivided into non-mixing cell populations called compartments that define lineage restriction boundaries. Compartment boundaries are remarkably straight and provide constituent cells with positional information and global orientation, serving as landmarks for tissue patterning and morphogenesis^1,2^. The signaling events that maintain boundaries during epithelial tissue morphogenesis have been intensively studied in the developmental compartments of *Drosophila*^3–8^. However, the precise molecular mechanisms that link intercellular signaling and physical cell sorting at the compartment boundaries have remained elusive.

Theoretical works have proposed distinct models to explain cell sorting in tissues or reconstituted tissues. One such proposal, the differential interfacial tension hypothesis, considers cortical tension to be a direct driving force for cell sorting^9,10^. In zebrafish, it has been shown that cortical contractility driven by Myosin II dominates the cell–cell adhesion force to determine the cell contact tension and therefore serves as the primary factor that propels cell sorting of the germ layer^11,12^. It has also been shown in *Drosophila* tissues that local increases in mechanical tension on cell junctions along the compartment boundaries counteract the mechanical challenges caused by cell proliferation and rearrangement^13–15^. These studies highlight the importance of cortical tension as a driving force to sort cells at tissue boundaries^16^. A longer-standing model, the differential adhesion hypothesis, focuses on the contrast in adhesive properties between two cell populations^17^ as the principal force behind cell sorting. This thermodynamic theory has been validated by numerical simulations but verified experimentally only in cultured cell systems^18–22^.

**Fig. 1:**
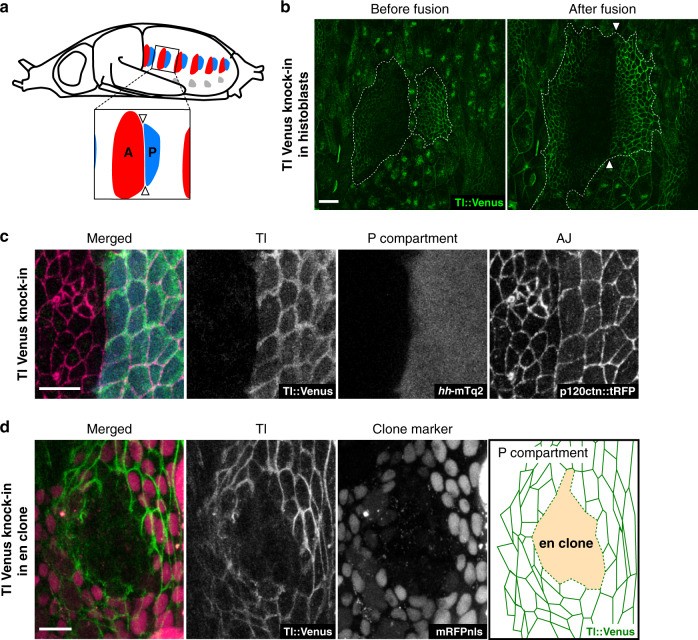
Toll-1 is expressed in P histoblasts under the control of *engrailed*. **a** Schematic depicting anterior (red) and posterior (light blue) histoblast nests in the epidermis of the *Drosophila* pupal abdomen. The boxed region highlights abdominal segment 2, and arrowheads point to the AP boundary. **b** Tissue wide expression pattern of Tl using fluorescently tagged endogenous Tl protein. Arrowheads point to the AP boundary. Anterior is to the left and posterior is to the right unless otherwise stated in this and all the following figures. Presented data are a representative image of *n* = 10 animals. Scale bar: 30 μm. **c** Tl protein localization visualized by Venus knock-in (green). Adherens junctions and P histoblasts were labeled with p120ctn::tagRFP (magenta) and *hh*::mTurquoise2 (cyan), respectively. Presented data are a representative image of *n* = 10 animals. Scale bar: 10 μm. **d** Mosaic analysis of Tl expression in *engrailed* (*en*) mutant cells. Tl expression was monitored with the Venus knock-in (green). Cells mutant for *en* were labeled with the loss of the marker expression, a monomeric red fluorescent protein with nuclear localization signal (mRFPnls) (magenta). Presented data is a representative image of *n* = 5 clones. Scale bar: 10 μm.

Although previous works have identified several differentially expressed adhesion molecules across boundaries, none has been shown to be responsible for cell sorting during tissue morphogenesis^23–25^. The *Drosophila* histoblasts, which consist of epithelial sheets of the epidermis in the abdomen, are subdivided into anterior (A) and posterior (P) compartments within each segment^26–28^. Cells of A and P compartments form discrete cell populations called histoblast nests embedded in the larval epidermal tissue (Fig. 1a). During the pupal stage, the histoblast cells proliferate and replace surrounding larval epidermal cells, resulting in the fusion of A and P histoblast nests. This fusion forms the sharp AP compartment boundary, which is then maintained throughout development. Cell sorting at the AP compartment boundaries requires the posterior specific selector gene *engrailed* and Hedgehog signaling transduction in P and A cells, respectively^4,5,28,29^. Although the local increase in mechanical tension on cell junctions along the AP boundary explains the biased intercalations that maintain straight boundaries in the proliferating tissue^14^, whether this change in tension is the only physical mechanism that shapes the straight boundary is unknown.

Here, we show that the differential adhesion system indeed works to maintain sharp compartment boundaries in the histoblasts of *Drosophila* abdominal epidermis. *Toll-1* is expressed across the entire posterior compartment under the control of the selector gene *engrailed* and displays a sharp expression boundary that coincides with the compartment boundary. *Toll-1* corrects local distortions of the boundary in the absence of cable-like Myosin II enrichment along the boundary. The reinforced adhesion of homotypic cell contacts, together with pulsed cell contraction, achieves a biased vertex sliding action by resisting the separation of homotypic cell contacts in boundary cells. Our data reveal how adhesion contributes to tissue morphogenesis.

## Results

### Sharp expression boundary of *Toll-1* at the compartment boundary is established by *engrailed*

Toll family receptor genes are expressed in stripes parallel to the segment boundaries during *Drosophila* embryogenesis and are required for the actomyosin-driven convergent extension of the germ band^30^. We asked whether *Toll* genes play a role in the maintenance of compartment boundaries, which also depends on the local regulation of the actomyosin cytoskeleton^31^. Expression of four out of nine *Drosophila* Toll family receptor genes was detected in the histoblasts of the pupal abdomen (Supplementary Fig. 1a, b). In order to examine their expression pattern, we generated Venus knock-in lines for these genes and found that *Toll-1* (*Tl*) is expressed in two domains: one in the A compartment and the other in the P compartment. While the anterior expression domain was observed only in the far-anterior region with a gradient increasing toward the anterior edge (Fig. 1b), the posterior expression was observed across the entire P compartment, displaying a sharp expression boundary at the AP boundary in the abdominal histoblasts (Fig. 1b, c). The sharp expression boundary coincided precisely with the compartment boundary (Fig. 1c). None of the other *Toll* gene knock-in lines showed a compartment-specific expression (Supplementary Fig. 1c). An expression reporter for *Tl* recapitulated the differential expression, suggesting transcriptional regulation of the *Tl* in the P compartment (Supplementary Fig. 1d, e). The posterior specific selector gene *engrailed* (*en*) is required for sorting P cells from A cells in the abdominal histoblasts as well as in the wing imaginal discs^4,28^. To examine whether the posterior expression of *Tl* is controlled by *en*, we induced somatic clones of mutant cells for *en* and its paralogue, *invected* (*inv*). The *en*/*inv* mutant clones generated in the P compartments displayed a round shape with a smooth border, as has been observed in wing imaginal discs (Fig. 1d)^5,14^. In these clones, Tl::Venus expression was abolished, suggesting the posterior compartment expression of *Tl* is induced by *en*. The anterior Tl::Venus expression was not affected in the *en*/*inv* mutant clones, showing that the anterior expression is regulated by a distinct mechanism from that of the P compartment (Supplementary Fig. 2a). Moreover, although *four jointed* (*fj*) displays apparently similar expression patterns to *Tl*^32^, neither the Tl::Venus expression nor its membrane localization was affected in *fj* mutant clones (Supplementary Fig. 2b). These results suggest that the *Tl* expression in the P compartment is regulated in an *en* dependent and *fj* independent manner.

### *Toll-1* is required for maintaining a straight AP compartment boundary

**Fig. 2:**
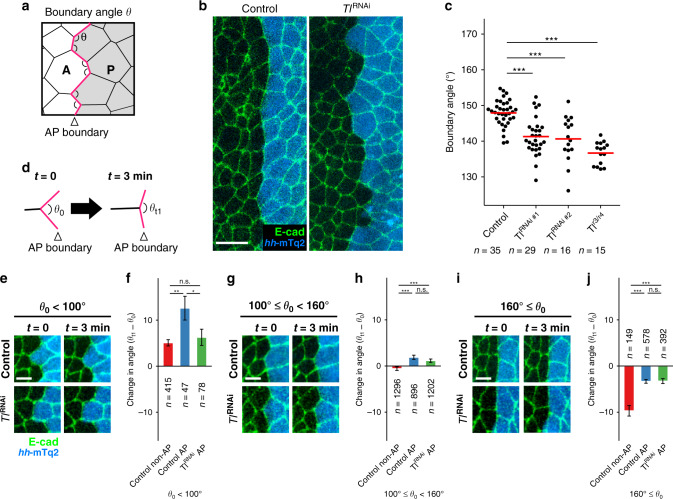
*Toll-1* is required for maintaining the sharp histoblast AP boundary. **a** Schematic representation of boundary angle measurement. The boundary angle *θ* is defined as the angle made by a pair of neighboring cell junctions on the AP boundary. **b** The AP boundaries in control (left, 23 hAPF) and *Tl* knockdown (right, 23 hAPF) histoblasts by expressing a short hairpin RNA for *Tl* using a histoblast-specific GAL4 driver, *esg*-GAL4. Scale bar: 10 μm. **c** Quantification of the boundary angle in control, *Tl* knockdown, and *Tl* mutant tissues as a readout for the sharpness of the AP boundaries. *n* = 35, 29, 16, and 15 boundaries for Control, *Tl*^RNAi #1^, *Tl*^RNAi #2^, and *Tl*^r3/r4^, respectively. Images were analyzed at the proliferation phase (22–24 hAPF). Statistical significances were evaluated using a Student’s *t* test (unpaired, two-sided). ***p < 0.001. **d** Diagram illustrating the measured boundary angle for the quantification of boundary angle change shown in (**e**–**j**). **e**–**j** Cellular configuration-dependent dynamics of the boundary angle change. Boundary angle (*θ*_1_) change over 3 min for the pair of junctions that form the boundary angle (*θ*_0_) less than 100˚ (**e**), between 100˚ and 160˚ (**g**), and more than 160˚ (**i**) 3 min before the measurement in the control and *Tl* knockdown tissues and the quantification of the boundary angle change (**f**, **h**, and **j**). Scale bars: 5 μm. Numbers of analyzed vertices (*n*) were indicated in graphs. Data were collected from two animals (22–24 hAPF, proliferation phase) for each experiment. Data are presented as mean values ± SEM. Statistical significance was evaluated using a Student’s *t* test (unpaired, two-sided). *p < 0.05, **p < 0.01, and ***p < 0.001.

We next asked whether the posterior compartment expression of *Tl* is required for maintaining the straight AP boundary in the histoblasts. The boundary angle along the AP boundary in the *Tl* knockdown tissue was significantly smaller than the control AP boundary (Fig. 2a–c). The same effect was obtained by a knockdown using a second short hairpin construct, as well as in a mutant allelic combination for *Tl*, confirming the specificity of the phenotype for *Tl* (Fig. 2c, Supplementary Fig. 3a). Although the AP boundary was straighter right after the fusion of A and P histoblast nests than it was at a later stage both in control and *Tl* RNAi tissues, the *Tl* RNAi boundary was already less straight compared to control at this time point (Supplementary Fig. 3b). During the phase at which histoblasts vigorously proliferate (proliferation phase, see Methods for the definition), the compartment boundaries were sometimes distorted locally at the single-cell scale. Such local distortions were under a strong bias to be smoothed out on the time scale of minutes, as shown by measuring the change in boundary angles for cells having initially small (less than 100°), intermediate (between 100° and 160°), and large boundary angles (more than 160°) (Fig. 2d). We asked whether the wavy boundaries observed in *Tl* knockdown tissues are defective in this immediate correction of the local distortions and found that the correction of the distortion was less effective in the *Tl* knockdown tissues. This result suggests that the wavy boundary observed upon *Tl* knockdown was due to the defect in the correction of the local distortion (Fig. 2e–j). Intriguingly, the cellular dynamics remained unchanged upon *Tl* knockdown when the AP boundary was not distorted (Fig. 2e–j).

### Differential expression of *Toll-1* is sufficient for smoothing boundaries, and *Toll-1* functions as an adhesion molecule

**Fig. 3:**
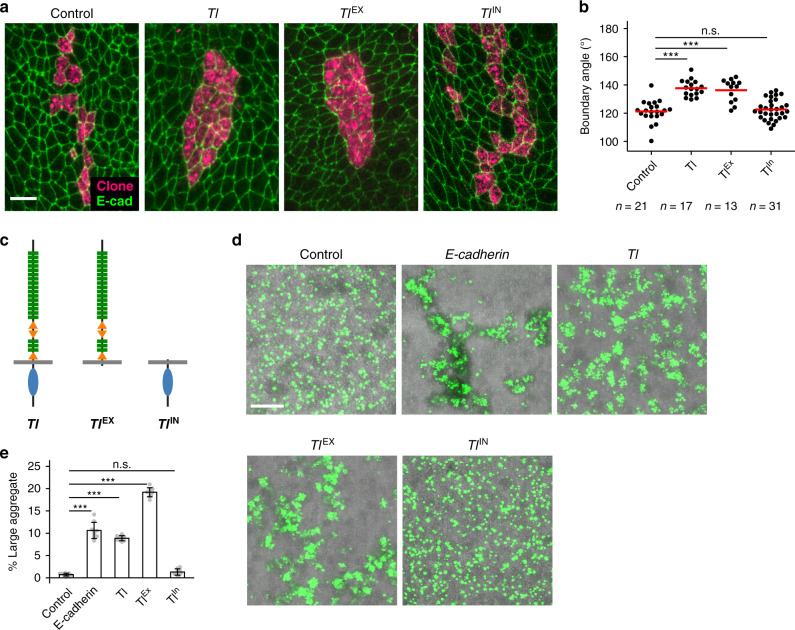
Expression of *Toll-1* makes cells adhere to each other. **a**–**c** Mosaic clones ectopically expressing *Tl* in the A histoblast nest. Expression of full length and the extracellular domain of *TI* renders clone contours smooth (**a**). The boundary angle of the *Tl* expressing clones for each experiment (**b**). Schematics of Tl full length, extracellular domain alone, and intracellular domain alone (**c**). *n* = 21, 17, 13, and 31 boundaries for Control, *Tl*, *Tl*^Ex^, and *Tl*^In^ overexpression clones, respectively. Statistical significances were evaluated using a Student’s *t* test (unpaired, two-sided). ***p < 0.001. Scale bar: 10 μm. **d** Overexpression of Tl results in cell aggregates. *Drosophila* S2 cells expressing E-cadherin (positive control), Tl full length (Tl), Tl extracellular domain (Tl^EX^), and intracellular domain (Tl^IN^). Scale bar: 100 μm. **e** Quantification of the aggregate formation. The fraction of the large aggregate was quantified. Data are presented as mean values ± SEM. Statistical significance was assessed using a Mann–Whitney *U* test (two-sided). *n* = 9 independent replicates for each experiment. *p < 0.05, **p < 0.01, and ***p < 0.001.

Next, we asked whether the differential expression of *Tl* is sufficient to form sharp boundaries. We found that the borders of the clones overexpressing *Tl* were straighter than those of control clones (Fig. 3a–c). The simplest interpretation of this phenotype would be that increased adhesion results in maximized homotypic cell–cell contacts. Although the involvement of *Drosophila* Tl, which contains leucine-rich repeat (LRR) motifs in the extracellular domain, has been suggested in the control of cell adhesion^33,34^, it is still possible that Tl controls cell adhesion via the regulation of downstream adhesion molecules, as has been reported for other LRR proteins^35^. Therefore, we asked whether the intracellular domain, which serves as a strong inducer of the downstream immune response through transcriptional activation^36,37^, is required for this activity. We found that ectopic expression of the extracellular domain but not the intracellular domain in somatic clones made the clone contour smooth within the A compartment (Fig. 3a–c). To confirm the activity of Tl as an adhesion molecule, non-adhesive *Drosophila* S2 cells were transfected with the Venus-tagged full-length, extracellular domain, or intracellular domain of Tl (Fig. 3c, d) and assayed for the formation of cell aggregates. The expression of either full length or extracellular domain Tl alone was sufficient to aggregate cell clumps to a similar extent as the overexpression of E-cadherin, a major adhesion molecule at the adherents junction^38^ (Fig. 3d, e, Supplementary Fig. 4a). The intracellular domain did not induce cell aggregation and was indistinguishable from negative control (Fig. 3d). Taken together, we concluded that Tl functions as an adhesion molecule in epithelial cell sorting, requiring the extracellular but not the intracellular domain.

### *Toll-1* functions as a homophilic adhesion molecule

Previous works have suggested that *Drosophila* Tl and some Toll receptors act as heterophilic adhesion molecules^30,34,39^. Our cell aggregation assay, however, indicates homophilic binding of Tl. As S2 cells express *Toll* genes (Supplementary Fig. 4b), we cannot rule out the possibility that Tl aggregates through heterophilic binding activity. Therefore, we decided to assess the homophilic binding activity of Tl. Tl::Venus knock-ins exhibited reduced Tl levels on cell junctions along the AP boundary and on the edges of *en* clones (Fig. 1c, d), suggesting that membrane localization of Tl may require *trans* binding with Tl presented by neighboring cells. When the cells overexpressing *Tl* were induced in the A compartment adjacent to the AP boundary, cell junctions that normally have less Tl::Venus became enriched with Tl::Venus (Supplementary Fig. 4c-d), indicating that *trans* binding of Tl is sufficient to recruit Tl at cell–cell contacts in adjacent cells. Consistently, Tl was absent at the edges of the clones that express Tl tagged with Venus while Tl localized at the cell–cell contacts within the clones (Supplementary Fig. 4e). Taken together, these experiments provide evidence that Tl localization on the cell membrane of epithelial cells is dependent on *trans* homophilic interaction, although it is worth noting that we cannot rule out the possibility that some ligand molecule(s) may be able to bridge the interaction.

### Differential expression of *Toll-1* does not induce Myosin II accumulation at the AP boundary

**Fig. 4:**
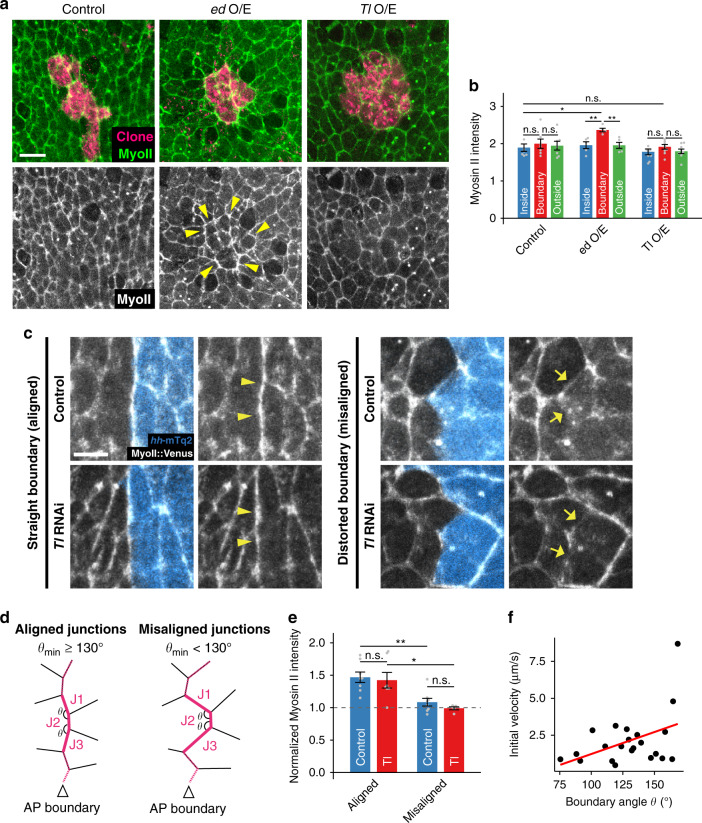
Toll-1 does not affect junctional Myosin II localization and its localization is largely unaffected by the actomyosin cytoskeleton. **a**, **b** Differential expression of *Tl* does not accumulate Myosin II at the interface between *Tl* expressing and non-expressing cells. While the expression of Ed in mosaic clones (magenta) accumulates junctional Myosin II (green) at clone edges, *Tl* expressing clones do not affect the junctional localization of Myosin II. Scale bar: 10 μm. Myosin II localization along the clone boundary (**b**). The signal intensity of endogenous Myosin II regulatory light chain tagged with the Venus fluorescent protein (Myosin II::Venus) on cell junctions was normalized to that of cytoplasm. 7, 5, and 9 clones were analyzed for control, *ed,* and *Tl* overexpressing clones, respectively. Data are presented as mean values ± SEM. Statistical significance was assessed with a Mann–Whitney *U* test (two-sided). *p < 0.05, **p < 0.01, and n.s. not significant. **c** Junctional Myosin II localization along the AP boundary when the boundary is straight or distorted. Myosin II::Venus (white) and *hh*::mTq2 (cyan). Myosin II enriched on cell junctions along the AP boundary when the boundary is straight (arrowheads) while the enrichment was not visible when the boundary was locally distorted in control experiments (arrows). Knockdown of *Tl* did not affect the enrichment of Myosin II::Venus when the boundary was straight. The AP boundary was visualized with the P histoblast-specific expression of *hh*::mTq2. Presented data are representative images of aligned junctions from *n* = 8 (control) and 6 (*Tl* RNAi) boundaries and misaligned junctions from *n* = 8 (control) and 5 (*Tl* RNAi) boundaries. Scale bar: 5 μm. **d**, **e** Schematic representation of aligned and misaligned junctions analyzed in (**c**, **d**). Three consecutive junctions (J1–J3) along the AP boundary (magenta) were analyzed for Myosin II signal intensity (see “Methods” for detail). Cases, where either of the boundary angles of the junction set was larger than or equal to 130˚, were considered as “aligned” and the smaller than 130˚ as “misaligned”. The relative intensity of Myosin II (normalized to that of non-boundary junctions) were plotted (**e**). The data were collected from staged pupae at the proliferation phase (21–24 hAPF). In total, 8 and 7 aligned junctions (from *N* = 8 and 6 boundaries) were analyzed for control and *Tl* RNAi, and 8 and 5 misaligned junctions (from *N* = 8 and 5 boundaries) were analyzed for control and *Tl* RNAi, respectively. Data are presented as mean values ± SEM. Statistical significance was assessed with a Mann–Whitney *U* test (two-sided). *p < 0.05, **p < 0.01, and n.s. not significant. **f** Relationship between the initial velocity after laser ablation of junctions on the AP boundary and boundary angle made between the ablated junction and its connected junction. Experiments were performed in the pupae at the proliferation phase (22–24 hAPF).

Local increases in Myosin II and mechanical tension at cell junctions along compartment boundaries are known to maintain the boundaries’ morphological integrity^13,14,40^. Moreover, differential expression of Toll family receptor genes is proposed to regulate local enrichment of junctional Myosin II at the expression boundaries^30^. We, therefore, asked whether differential expression of *Tl* induces the accumulation of actomyosin cables at clone edges. In contrast to the overexpression of the adhesion molecule echinoid (ed), which results in the recruitment of Myosin II forming actomyosin cable spanning multiple cell junctions^41,42^ (Fig. 4a, b), cell junctions at the border of *Tl* overexpressing clones were not enriched with junctional Myosin II (Fig. 4a, b). This result prompted us to examine whether *Tl* regulates cell sorting at the compartment boundary without locally increasing junctional Myosin II. To do so, we analyzed Myosin II localization when the boundary was locally distorted, during which *Tl* is responsible for maintaining the straight boundary. Stable multicellular cable-like Myosin II accumulations were observed at the cell junctions along the compartment boundary when the boundary was straight (Fig. 4c–e, Supplementary Fig. 5a). However, the cable-like local enrichment was hardly observable when the compartment boundary was locally distorted (Fig. 4c–e, Supplementary Fig. 5a). Moreover, cell junction tension, probed by measuring the recoil of vertices after laser ablation, showed a positive rather than negative correlation with the boundary angle, suggesting no active response to the local distortion of the boundary (Fig. 4f). Taken together, these results confirm that neither Myosin II nor tension on cell junctions along the AP boundary is actively increased in response to local distortion. Consistent with this result, Myosin II accumulation along the AP boundary was unaffected by *Tl* knockdown (Fig. 4c–e), confirming that *Tl* maintains the straight boundary with a mechanism acting in parallel to the cable-like accumulation of junctional Myosin II. We also observed that Myosin II accumulation, which was prominent on cell junctions along the AP boundary initially after the fusion of A and P compartment histoblasts, faded over time and became barely detectable, suggesting the relaxation of mechanical tension on cell junctions along the AP boundary during the high histoblast proliferation period (Supplementary Fig. 5b, c).

Unlike the adherens junction components^43–45^, Tl localization at cell–cell contacts was still observed despite the presence of the Rok inhibitor, which severely affects the localization of adherens junction components, suggesting that the membrane localization of Tl is mechanistically distinct from the assembly of the adherens junctions and could therefore act independently from the adherens junction in adhering cells (Supplementary Fig. 5d).

### Pulsed contraction of apical cell area provides a driving force for the correction of local boundary distortions

**Fig. 5:**
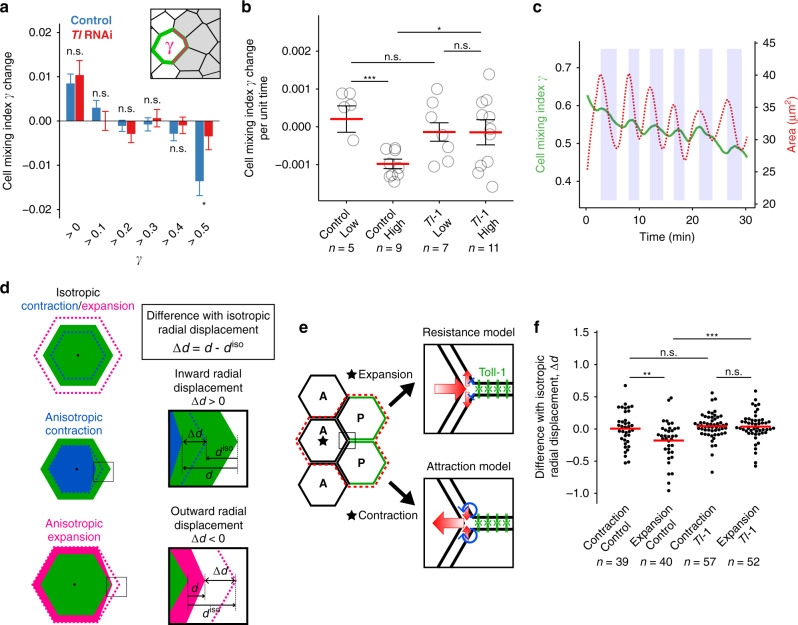
*Trans* interaction of Toll-1 between P histoblasts increases homotypic cell contacts by biasing the vertex sliding. **a** The reduction of cell mixing index γ at the locally distorted AP boundary is dependent on *Tl*. Cell mixing index *γ* is the fraction of the adherent’s junctional length of cells that are in contact with cells of the adjacent compartment. Change in *γ* between two frames (3 min) was measured for individual cells categorized by the value for *γ* at the first frame. Totally, 1220 cells (107, 240, 346, 251, 211, and 65 cells for >0, >0.1, >0.2, >0.3, >0.4, and >0.5 bins, respectively) from 6 boundaries for control and 964 cells (91, 176, 204, 241, 189, 63 cells for >0, >0.1, >0.2, >0.3, >0.4, >0.5 bins, respectively) from 5 boundaries at the proliferation phase (21–23 hAPF) were analyzed. Data are presented as mean values ± SEM. *p* Values were from the Student’s *t* test (unpaired, two-sided). *p < 0.05. **b** Cells with higher area fluctuation reduce *γ* efficiently in control but not in *Tl* knockdown tissue. The analysis was performed for A cells having *γ* > 0.5 (10 animals for control and 15 animals for *Tl* knockdown at the proliferation phase (21–23 hAPF)). Cells were categorized as low or high fluctuation based on the degree of area fluctuation (see “Methods”) and were analyzed for *γ* change separately. Statistical analysis was performed using the lm function in R (version 4.0.0). The number of analyzed cells were indicated as *n*. Data are presented as mean values ± SEM. Statistical significance was determined using a Mann–Whitney *U* test (two-sided). *p < 0.05, ***p < 0.001, and n.s. not significant. **c** The cell mixing index *γ* declines as the area of the cell fluctuate. The cross-sectional area and γ for an A cell that has initially high γ were plotted as a function of time. **d** Measurement of vertex sliding productivity at the AP boundary during contraction and expansion. The two possible situations that would result in the straightening of the AP boundary are illustrated. The productivity of vertex displacement was evaluated by comparing cells’ displacement behavior to the hypothetical vertex displacement that they would undergo if the cell exhibited isotropic cell shape change with the same degree of area change (Δ*d*). Δ*d* is calculated by subtracting the calculated displacement of the vertex (*d*^iso^) when the isotropic contraction was supposed from the actual displacement (*d*) of the vertex on the AP boundary for A cells for the expansion phase and contraction phase, separately. **e** Two models illustrating the homophilic interaction of Tl regulating vertex sliding at the interface between A and P histoblasts. The analyzed cell configuration is shown on the left (one A cell and two P cells, highlighted with red dashed line). Large red arrows indicate the direction of vertex sliding; small red arrows indicate the direction of the forces exerted on the cell–cell contact between P cells, which result in either separation (top) or closure (bottom) of homotypic cell–cell contacts between P cells. Blue arrows represent the recruitment of more Tl at the leading edge of the cell–cell contact between P cells. **f** *Tl* was required for the biased outward vertex sliding for A cells at the AP boundary. Δ*d* was plotted for cells that initially had a high *γ* value (*γ* > 0.55) at each area expansion and contraction phase separately (three cells each for control and *Tl* knockdown) from the pupae at the proliferation phase (21–23 hAPF). Outward sliding was less effective than expected from hypothetical isotropic vertex displacement in controls. Inward sliding was comparable to the hypothetical isotropic vertex displacement. The outward vertex sliding became unbiased in the *Tl* knockdown tissue. The numbers of analyzed phases (contraction or expansion) were indicated as *n*. Statistical significance was determined using a Student’s *t* test (unpaired, two-sided). **p < 0.01, and ***p < 0.001.

In order to gain insight into the cellular and molecular mechanisms for cell sorting regulated by differential expression of Tl and acting in parallel with local enrichment of junctional Myosin II, we quantitatively analyzed cell dynamics when the AP boundary was locally distorted. When boundary cells have a large fraction of cell contacts with cells of the opposite compartment, they show a strong bias to move back to their original compartment^14^ (Cell mixing index *γ* > 0.5; Fig. 5a). This bias was significantly reduced in the *Tl* knockdown tissues, consistent with our finding that *Tl* is required for correcting local distortions (Fig. 5a, Fig. 2e, f). Epithelial cells commonly exhibit pulsed contraction of cell area driven by the apicomedial actomyosin network, a distinct actomyosin population from junctional actomyosin^46^. The apicomedial Myosin II population in the histoblasts also exhibited pulsed centripetal dynamics, and those dynamics were unaffected by *Tl* knockdown (Supplementary Fig. 6a, b; Movies 1 and 2). The pulsed apicomedial Myosin II coalescence regulates the contraction and relaxation of apical cell area and drives the apical constriction of the cells during *Drosophila* embryogenesis via ratchet-like mechanisms^47–49^. We, therefore, examined a possible functional link between the fluctuation of the cell area and the correction of local distortions. Given that Tl proteins are present at cell junctions between P cells, we decided to focus our analysis on the dynamics of the protruding A cells (see Fig. 5e, left) since the elongation of cell–cell contact between a pair of P cells at the AP boundary would push back the protruding A cell by sliding a vertex at the AP boundary toward the A compartment. The contraction/expansion dynamics of the A cell should provide a greater contribution to vertex sliding than that of the pair of P cells in this configuration due to the cellular geometry^50–52^. The protruding A cells displaying high area fluctuation, but not low area fluctuation, were under a bias to reduce the cell contact with the cells from the P compartment, suggesting that the area fluctuation has a positive effect on correcting local distortion (Fig. 5b, c). This positive effect on cell sorting requires *Tl* function since the *Tl* knockdown abrogated the bias to reduce the cell mixing index for the cells with high area fluctuation (Fig. 5b). The degree of area fluctuation was unchanged in *Tl* knockdown, consistent with the idea that Tl acts in parallel with Myosin II (Supplementary Fig. 6c, d). Taken together, the area fluctuation of A cells promotes the retraction of A cells only in the presence of Tl expressed in P cells, suggesting that area fluctuation provides a driving force for the correction of the local boundary distortion.

### *Toll-1* resists the separation of homotypic cell–cell contacts

The reduction of cell contacts with opposite compartment cells suggests that the boundary cells undergo anisotropic cell shape change during the correction (Fig. 5c, Movie 3). This anisotropic cell shape change at the AP boundary represents the difference in effective contraction and/or expansion of on-boundary junctions versus off-boundary junctions for boundary cells. The centripetal movement of apicomedial actomyosin coalescence within a cell exerts pulling forces on the vertices, causing them to slide inward and thereby contracting the apical cell area^50^. If the vertices on the AP boundary of a boundary cell slide differentially from the rest of the cell, the boundary cell would undergo the observed anisotropic cell shape change. Therefore, we decided to quantitatively analyze vertex sliding at the AP boundary (Fig. 5d, Supplementary Fig. 6e, f). When an A cell happened to protrude into the P compartment, the vertices located at the AP boundary would undergo more inward (anteriorward) than outward (posteriorward) displacement, thereby correcting the local distortion. There are two possible mechanisms that could account for such an anisotropic vertex displacement: first, less efficient outward displacement than the other vertices of the cell during expansion (resistance model, Fig. 5e, Supplementary Fig. 6h) or, second, more efficient displacement during contraction (attraction model, Fig. 5e, Supplementary Fig. 6h). The radial displacement of the vertices on the AP boundary was also pulsatile, suggesting that the motion of boundary vertices are also coupled with area contraction^50^ (Supplementary Fig. 6g). This pulsating vertex radial displacement was still observed in the *Tl* knockdown tissue, suggesting that *Tl* is dispensable in the transmission of contractile forces to the vertices (Supplementary Fig. 6g; Movie 4). To examine if either the resistance model or the attraction model could explain the correction of the local distortions, we analyzed vertex displacement for A cells that happened to protrude into the P compartment during the area expansion and contraction phases separately (Fig. 5d–f). We defined Δ*d* as a difference of actual vertex displacement from hypothetical isotropic radial displacement. If Δ*d* is positive during a pulse of contraction (more vertex displacement toward the cell center than that of hypothetical isotropic contraction) or negative during a pulse of expansion (less vertex displacement away from the cell center than that of hypothetical isotropic expansion), then the boundary will become straighter as a result. It should be noted that the Δ*d* can also take negative values during contraction and positive values during expansion. The vertices at the AP boundary were displaced significantly less than when the cell would undergo isotropic cell shape change during expansion phases (Fig. 5d–f). This outward displacement bias was abolished in the *Tl* knockdown tissues, suggesting that the Tl *trans* homophilic adhesions resist the sliding of vertices that would otherwise cause the separation of homotypic cell–cell contacts (Fig. 5e, f). Inward vertex displacements at the AP boundary were comparable to those of isotropic cell shape change during the contraction phase, both in control and *Tl* knockdown (Fig. 5f). These results suggest that the adhesion mediated by Tl contributes to extending the homotypic cell–cell contacts between P cells by resisting the separation of these junctions.

## Discussion

It is now commonly recognized both in *Drosophila* and zebrafish tissues that a local increase in Myosin II and mechanical tension at compartment boundaries are a major driving force to maintain their sharp, straight morphology^13–15,40,53^. Our data suggest that differential adhesion implemented by the differential expression of Tl regulates cell sorting at the compartment boundary independently of a junctional Myosin II-based mechanism. It is proposed that increased cell–cell adhesion reduces interfacial energy and thereby contributes to cell contact increase^54–60^. Tl homophilic adhesion primarily increases homotypic cell contacts and consequently minimizes the heterotypic cell contacts in the epithelium (Supplementary Fig. 6e, f). The question then is how adhesion molecules increase cell contacts. There are two possible scenarios for the underlying mechanism, which are (1) the resistance model and (2) the attraction model (Fig. 5e, Supplementary Fig. 6h). In the resistance model, homophilic adhesion of Tl resists the outward movement of the vertex at the AP boundary in the cell expansion phase of protruding P cells, since the corruption of homotypic adhesion between P cells, which are reinforced by Tl, would require more energy than that of heterotypic ones. On the other hand, in the attraction model, homophilic adhesion facilitates the inward movement of the vertex in the cell contraction phase, as the homophilic adhesion actively brings the opposing cell surfaces together by attracting its cohort molecule on the opposing cell. This activity would form a positive feedback loop with the contractile forces exerted on the vertex and would promote the elongation of homotypic cell–cell contacts at the expense of heterophilic cell–cell contacts, as if they were actively zipping up the homotypic cell–cell contacts. Both models would result in the same outcomes: a net inward vertex displacement and the correction of local distortions for an A cell (Fig. 5d, e, Supplementary Fig. 6h). In mouse blastocysts, increased E-cadherin levels lead to the increase of cell–cell contact area resulting in compaction^61–63^. The cells actively drive this process by extending actin-based protrusions onto the surface of neighbor cells^64^. Our quantification of the vertex motion in the *Tl* knockdown tissue contradicts the presence of such an active process in increasing the homotypic cell contact area in the histoblasts. Instead, the result suggests that the contribution of adhesion in increasing cell contacts is passive, therefore supporting the resistance model in which adhesion molecules act as a scaffolding link between neighboring cells. In many cases, the adhesion strength provided by cadherins is not sufficient to increase the contact area and overcome the cortical tension of the cells but rather merely links neighboring cells with very limited contact^16,59,65,66^. The actomyosin-independent feature of Tl enables cells to uncouple the control of adhesion from the regulation of the actomyosin cytoskeleton. Increasing cell–cell adhesion without affecting actomyosin subcellular organization would benefit cells by allowing them to reserve control of cortical contractility for other cell activities such as pulsed contraction or tissue integrity maintenance in making A or P cells indistinguishable from each other during morphogenesis. It is noteworthy that *en* maintains the compartment boundary with Hh signaling-dependent as well as -independent pathways in the *Drosophila* wing imaginal disc^5,67^. Notably, the Hh independent pathway regulates cell sorting without the local increase in Myosin II^68^. Our results showing *Tl* is regulated by *en* and that *Tl* regulates cell sorting in parallel with local increase of junctional Myosin II imply that *Tl* is responsible for the Hh independent *en* function in cell sorting. Further analysis will be required to evaluate this hypothesis.

Toll family receptor genes encode transmembrane proteins that possess LRR motifs at the extracellular domain and the TIR domain that is required for signal transduction at the intracellular domain^69,70^. The function of the mammalian homologs of Toll family receptor proteins, the so-called Toll-like receptors (TLR), as innate immunity sensors that recognize pathogen-associated molecular patterns has been well characterized^69^. By contrast, the non-immunity functions of the Toll family receptor proteins and the mammalian TLR, especially as an adhesion molecule, are much less understood. *Drosophila* Toll-2, -6, -8 receptors cooperatively regulate anisotropic contraction of transverse junctions to drive directed cell intercalations during germband elongation. *trans* heterophilic interaction between these Toll proteins is thought to be a key function in the regulation of this stage of morphogenesis. Interestingly, Toll-2, -6, -8 genes together with Toll-7 are structurally similar and comprise the so-called Long-Toll clade of proteins, which bear more LRR motifs than the rest of Toll family receptor proteins in insects^71^. Another work has also shown that all of the Long-Tolls are functionally replaceable in the context of neuronal targeting^39^. *Tl* does not belong to the Long-Toll clade and is the only gene that exhibits sharp expression boundary at the AP boundary among all the *Drosophila* Toll family receptor genes that are expressed in the histoblasts. Given that Tl has divergent molecular and functional characteristics in the form of homophilic adhesion and actomyosin independence, this structurally distinct protein may have been adopted to play a role in development that is different from that of the Long-Toll proteins, such as static versus active morphogenetic processes. This compelling possibility raises the stakes to better characterize the underrepresented non-immunity functions of Toll proteins, and we look forward to future works that will further this line of inquiry, especially in other tissues and organisms.

## Methods

### Fly stocks

The following fly stocks were used: *Tl*::Venus (this work)*, Toll-2*::Venus (this work)*, Toll-7*::Venus (this work)*, Toll-8*::Venus (this work), UAS-*Toll-1*^*FL*^ (this work), UAS-*Toll-1*^*INT*^ (this work), UAS-*Toll-1*^*EX*^ (this work), *hh-*mTurquoise2 (this work), *sqh*::Venus^72^, *hs-*flp; *Act5c* > CD2 > GAL4 UAS-CD8::mCherry (this work), UAS-*Toll-1*::Venus (this work)*, Toll-1* shRNA #1 (this work), *en-*GAL4 UAS-H2B::ECFP (this work)*, hh-*GAL4 UAS-CD8::mCherry (this work), *Toll-1*^*Mi{MIC}MI01254*^ (BL36134)*, Toll-1* shRNA #2 (BL31477), FRT42D *ubi-*mRFPnls (BL35496)*, nos-*φC31; attP40 (BL25709), *Tl*^r3^ (BL3238)*,Tl*^r4^ (BL2507) from Bloomington Stock Center, CAS-0001, CAS-0003 from NIG-FLY, *esg*-GAL4 (104863, DGRC Kyoto), UAS-*Toll-1*^73^, *en*^*E*^^74^, FRT42D *fj*^*d1*^^75^ (Dr. David Strutt), *en*-Venus^76^, *sqh-p120ctn*::TagRFP^77^, *DE-cad*::GFP^78^, and UAS-ECFP::Venus^79^. Flies were raised at 25˚C on standard food.

### Live image acquisition

White pupae were collected and incubated at 25 °C for 20–26 h before imaging, appropriately depending on experiments. For staged pupae, the pupal case was carefully removed on top of abdominal segments 1–4. The animals were placed on a coverslip, and images were acquired on a Leica TCS-SP8 Confocal Microscope (×63 GLY lens), a Leica TCS-SP5 inverted confocal microscope (×63 GLY lens, ×10 DRY lens), a Leica TCS-SP5 upright confocal microscope (×63 OIL lens), or a Zeiss LSM 880 confocal microscope (×40 OIL lens). Images were acquired at time intervals of 15 s, 3 min, or 5 min depending on experiments.

### Histoblast cell collection by FACS

To examine the Toll receptor family gene expression in abdominal histoblasts by reverse transcription-polymerase chain reaction (RT-PCR), the histoblasts were collected via fluorescence-activated cell sorting (FACS). To do so, 10–15 pupae (*esg-*GAL4, UAS-ECFP::Venus) at 26 APF were dissected and washed in phosphate-buffered saline (PBS) to obtain the abdominal epithelium containing histoblast cells. The samples were transferred into 200–500 μl of 0.25% Trypsin–EDTA, incubated for 30 min at room temperature and then dissociated into single cells by pipetting. After centrifugation at 100×*g* for 5 min at 4 °C, the cells were washed in 1% bovine serum albumin (BSA)/PBS and resuspended in 800 μl 1% PBS/BSA. Using S3eTM Cell Sorter (BIO RAD), fractions of Venus-negative and -positive cells were sorted and collected.

### RT-PCR analysis

Total RNA from the cells collected by FACS as described above was prepared using Direct-Zol^TM^ RNA MiniPrep (ZYMO RESEARCH), followed by a reverse transcription using PrimeScript RT reagent Kit with gDNA Eraser (TaKaRa). RT-PCRs were performed using primers listed in the primer and oligo DNA list provided as a Supplementary Table.

### Genomic DNA extraction

A single fly in a 1.5 ml tube was mashed for 5–10 s with a yellow pipette tip containing 50 μl of SB (Squishing Buffer: 10 mM Tris-HCl pH 8, 1 mM EDTA, 25 mM NaCl, 200 μg/ml Proteinase K). After incubating at 37 °C for 30 min and deactivating the Proteinase K by heating to 95 °C for 3 min, the supernatant was collected after brief centrifugation at 800×*g* for 3 min at 4 °C to obtain genomic DNA.

### Molecular biology

To construct gRNA plasmids for CRISPR/Cas9, the pBFv-U6.2 vectors were digested with *Bbs*I and ligated with the double-stranded oligo DNA sequences listed in the primer and oligo DNA list provided as a Supplementary Table.

For knock-in construct plasmids, the pBlueScriptII SK + vector was digested with *Eco*RI, and then ligated with a cassette containing the fluorescent protein Venus sequence excised from the pPVxRF3 plasmid or a *Drosophila* codon-optimized mTurquoise2 fluorescent protein from the pPTxRF3 plasmid with *Esp*3I and homologous recombination (HR) arms by the In-Fusion HD kit (Clontech). HR arms were amplified by PCR from genomic DNA extracted from a single CAS-0001 (NIG-FLY) adult fly. DNA constructs for *Toll-1*, *-2*, *-7*, *-8*, and *spaghetti squash* (*sqh*) knock-in were designed to insert a knock-in cassette containing the full-length Venus sequence into the site in front of the termination codon of each gene. The DNA construct for the *hh*::mTurquoise2 line was designed to insert a knock-in cassette containing the full-length mTurquoise2 sequence into the site behind the start codon of the *hedgehog* gene coding sequence using the same protocol for *Toll* gene knock-in constructs described above. PCRs were performed using the primers listed in the primer and oligo DNA list provided as a Supplementary Table.

To construct UAS-*Toll-1*^FL^, UAS-*Toll-1*^Ex^, and UAS-*Toll-1*^Int^ plasmids, *Toll-1* sequences were amplified from a cDNA library prepared from total RNA extracted from histoblasts using the primers listed in the primer and oligo DNA list provided as a Supplementary Table.

To construct UAS-*Toll-1*^FL^::Venus, UAS-*Toll-1*^Ex^::Venus, and UAS-*Toll-1*^Int^::Venus plasmids, pJFRC81-10xUAS-GFP was digested with *Kpn*I/*Xba*I and ligated with corresponding sequences for each Toll-1 construct and Venus. *Tl* sequences were amplified from a cDNA library prepared from total RNA extracted from histoblasts, and the Venus sequence was amplified from the plasmid pPVxRF3 using the primers listed in the primer and oligo DNA list provided as a Supplementary Table.

The generation of an shRNA construct for *Tl* followed a protocol previously described^80^. Briefly, a 22 base oligo DNA targeting the *Tl* gene was designed and concatenated 3 times. The obtained three tandem shRNA sequence was cloned to the pCM43b-UAS plasmid, digested with *Eco*RI/*Not*I. The primers used are listed in the primer and oligo DNA list provided as a Supplementary Table.

### Generation of transgenic flies

To generate knock-in strains using CRISPR/Cas9, 200 ng/μl gRNA plasmids, and 200–500 ng/μl plasmids containing knock-in constructs were prepared for the injection solution. To generate UAS-Toll-1-domain strains via P elements, 250 ng/μl plasmids were prepared. *nos*-Cas9 flies (CAS-0001, CAS-0003 from NIG-FLY) and *nos*-phiC31; attP40 flies were injected as early embryos to generate the transgenic strains.

### S2 cell aggregation assay

S2 cells were maintained in Schneider’s *Drosophila* Medium (ThermoFisher Scientific Gibco) containing 62.5 U/ml penicillin, 62.5 μg/ml streptomycin, and 10% FBS at 20 °C. For an S2 cell aggregation assay, 1.0 × 10^6^ cells were added to 1.6 ml medium in a 3.5 cm glass-bottom dish (Greiner Bio-One). After overnight incubation, plasmids for *Actin*-GAL4 and each UAS-*Toll-1* construct were transfected to the cells with the Effectene Transfection Reagent (QIAGEN). Two days later, after 10 min of gentle agitation at room temperature, cell aggregations were observed using a Leica TCS-SP5 inverted confocal microscope (×10 DRY, 3 × 3 tile scan). For the quantification, the aggregation sizes were obtained using Analyze Particles (ImageJ) after image thresholding. Objects larger than 25 px (image resolution: 0.66 px/μm) were considered to be a cell or a cell aggregate, and then the fraction of large aggregates whose size was larger than 1000 px was calculated. Statistical significance was assessed using the Mann–Whitney *U* test.

### Clonal analysis

Flies with the genotype *hs*-flp*; Act5c* > CD2 > GAL4 were subjected to a heat shock for 10 min at 37 °C at 2 days after egg lay and were dissected at 22–24 h after puparium formation. Flies with the genotype *hs*-flp; FRT42D *en*^*E*^/FRT42D *ubi*-mRFPnls or *hs*-flp; FRT42D *fj*^*D1*^/FRT42D *ubi*-mRFPnls were subjected to a heat shock for 90 min at 37 °C at 3rd instar larval stage and were dissected at 22–24 h after puparium formation. Prepared pupae were imaged immediately after dissection.

### Staging of pupa

In order to time the developmental stages of pupal specimens, we used the sensory organ precursor (SOP) development as a reference. The fusion of the A and P histoblast nests occurs about 2–6 h before the emergence of the first row of SOP development, which is highly reproducible in control, 23.0 ± 0.42 (SEM) hours after puparium formation (hAPF). *Tl* RNAi did not affect the developmental speed judged by this criterion (23.4 ± 0.45 hAPF, *p* = 0.56, Mann–Whitney’s *U* test). Since vigorous cell proliferation was observed around this time point, the time window ±2 h to the emergence of the first row SOPs was defined as the proliferation phase (21–25 hAPF). Subsequently, histoblasts begin to elongate along the dorsoventral axis, and this phase was defined as the “elongation” phase (27–29 hAPF). The “fusion” phase was defined for individual specimens by visual inspection for the fusion of A and P histoblast nests (17–20 hAPF).

### Boundary angle analysis

To quantify boundary angles in still images, coordinate information of cell vertices on the boundaries was obtained using ImageJ, and the angles between the cell vertices were calculated by custom code written in R (Comprehensive R Archive Network). In the quantification of boundary angles in movies, the angles between cell vertices on the AP boundaries were quantified using the ImageJ angle measure function. The boundary angle measurements at the AP boundary were performed between 22 and 24 hAPF for both control and *Toll-1* RNAi animals. Statistical significances were evaluated using Student’s *t* tests.

### Quantification of Myosin II signal intensity

To quantify Myosin II signal intensity along the AP boundary, the signal intensity of all junctions along the AP boundary and junctions one cell row away from the AP boundary on both sides were measured separately using the ImageJ line selection tool. Myosin II enrichment was calculated as the relative average signal intensity normalized by the average of Myosin II signal intensity of non-AP boundaries on each side of the AP boundary. To determine whether the multicellular Myosin II cable was formed, three connected junctions along the AP boundary were selected and quantified for the Myosin II signal intensity from histoblasts of staged pupae. The signal intensity on-boundary junctions were separately measured for each junction as a mean intensity on a 3-pixel wide line drawn on the junction. The obtained values were normalized to the mean signal intensity of ten junctions of the analyzed cells, excluding the boundary junction themselves. For the detection of multicellular Myosin II cables, the average signal intensity for three consecutive junctions (J1–J3 in Fig. 4d) along the AP boundary was analyzed. The Myosin II signal intensity assessments at the AP boundary were performed between 22 and 24 hAPF for both control and *Tl* RNAi animals.

### Quantification of Tl::Venus recruitment at the AP boundary next to overexpression clones

For quantification of Tl::Venus intensity on cell junctions at the AP boundary, the signal intensity on the boundary junctions was normalized to that of non-boundary cells for each cell located at the boundary. The signal intensity on junctions at the boundary and non-boundary was separately measured as a mean intensity on a 7-pixel wide line.

### Quantification of cell and vertex dynamics

For the quantitative analyses of cell and vertex dynamics, image segmentation, and cell tracking for images taken from E-cadherin::GFP flies were performed using the ImageJ plugin Tissue Analyzer^81^. The cell mixing index was calculated as has been reported previously^14^. For time-series analysis, obtained time-series values of area, cell mixing index, and vertex coordinate for each cell were smoothed using the Savitzky–Golay filter. The pulsed contraction was analyzed by detecting local minima and maxima for the cell area using a custom R code based on the calculation of the second derivative. The periods between local minima and maxima were defined as an expansion phase, and the others were deemed a contraction phase. To analyze the relationship between the cell mixing index *γ* change per unit time and the degree of area fluctuation (Fig. 5b), the long-term trend of *γ*, either increase or decrease, was estimated by fitting a linear model to the time series of *γ* in a 15 min time window for each cell. Cell junctions were auto-segmented followed by manual corrections and auto-cell tracking in the Tissue Analyzer. P cells with the maximum *γ* > 0.5 in the 15 min time window were analyzed. The degree of area fluctuation was defined as the product of the frequency and the sum of the absolute area change between local minima and maxima of the area within the time window. Cells with a degree of area fluctuation of greater than 0.6, normalized by the time average of their area, were categorized as high fluctuation cells, while those equal to or less than 0.6 were deemed low fluctuation cells. The productivity of vertex displacement (Fig. 5f) was evaluated by calculating Δ*d* during the expansion phase and the contraction phase, separately. Δ*d* was calculated by subtracting the calculated displacement of the vertex (*d*^iso^) during isotropic contraction, which was calculated from area change, from the actual displacement (*d*) of the vertex on the cell. Statistical analyses were performed using R.

### Calculation of virtual isotropic vertex displacement

Cell outlines obtained by auto-segmentation were used for the analysis. We first calculated the ratio of expansion/contraction of cell area between time frames. Then the square root value of the obtained ratio was used as a scaling factor to shift each vertex of the cell in radial orientation to obtain the *xy* coordinate for a virtual isotropic vertex displacement.

### Laser ablation analysis

Laser ablation experiments were performed using the MicroPoint system (Oxford Instruments Andor) mounted on a Lecia SP5 inverted confocal microscope. Images were taken every 500 ms after ablation. The vertex displacement after laser ablation was analyzed with ImageJ. The two vertices of the ablated cell junctions were tracked manually^14^.

### Drug injection in pupae

The Rok inhibitor Y-27632 was applied to live pupae by microinjection using a manual injection manipulator (NARISHIGE, Japan) and the FemtoJet 4i (Eppendorf, Germany). An aliquot of 25 mM Y-27632 was injected into the abdominal body cavity under a stereomicroscope, after which the injected animals were immediately imaged using the confocal microscope.

### Reporting summary

Further information on research design is available in the Nature Research Reporting Summary linked to this article.

## Supplementary information

Supplementary Information

Description of Additional Supplementary Files

Supplementary Movie 1

Supplementary Movie 2

Supplementary Movie 3

Supplementary Movie 4

Reporting Summary

## Data Availability

The data that support all experimental findings of this study are available within the paper and its Supplementary Information files or from the corresponding author D.U. upon request. Source data are provided with this paper. Raw data necessary to reproduce all statistical analyses and results in the paper as well as *P* values for all figures are provided in the source data file. Source data are provided with this paper.

## Source data

Source Data

## Code availability

R (version 4.0.0) codes were used for automated data analyses and data plotting. All codes are available on request from the corresponding author D.U.
