# Supplementary material for: Differential cell adhesion implemented by Drosophila Toll corrects local distortions of the anterior-posterior compartment boundary
Source: Nat Commun. 2020 Dec 10;11:6320. doi: 10.1038/s41467-020-20118-y (PMC7729853; doi:10.1038/s41467-020-20118-y)
Supplement: Supplementary file 1 — Supplementary Information [file 41467_2020_20118_MOESM1_ESM.pdf]

Supplementary Figures

Supplementary Figure 1

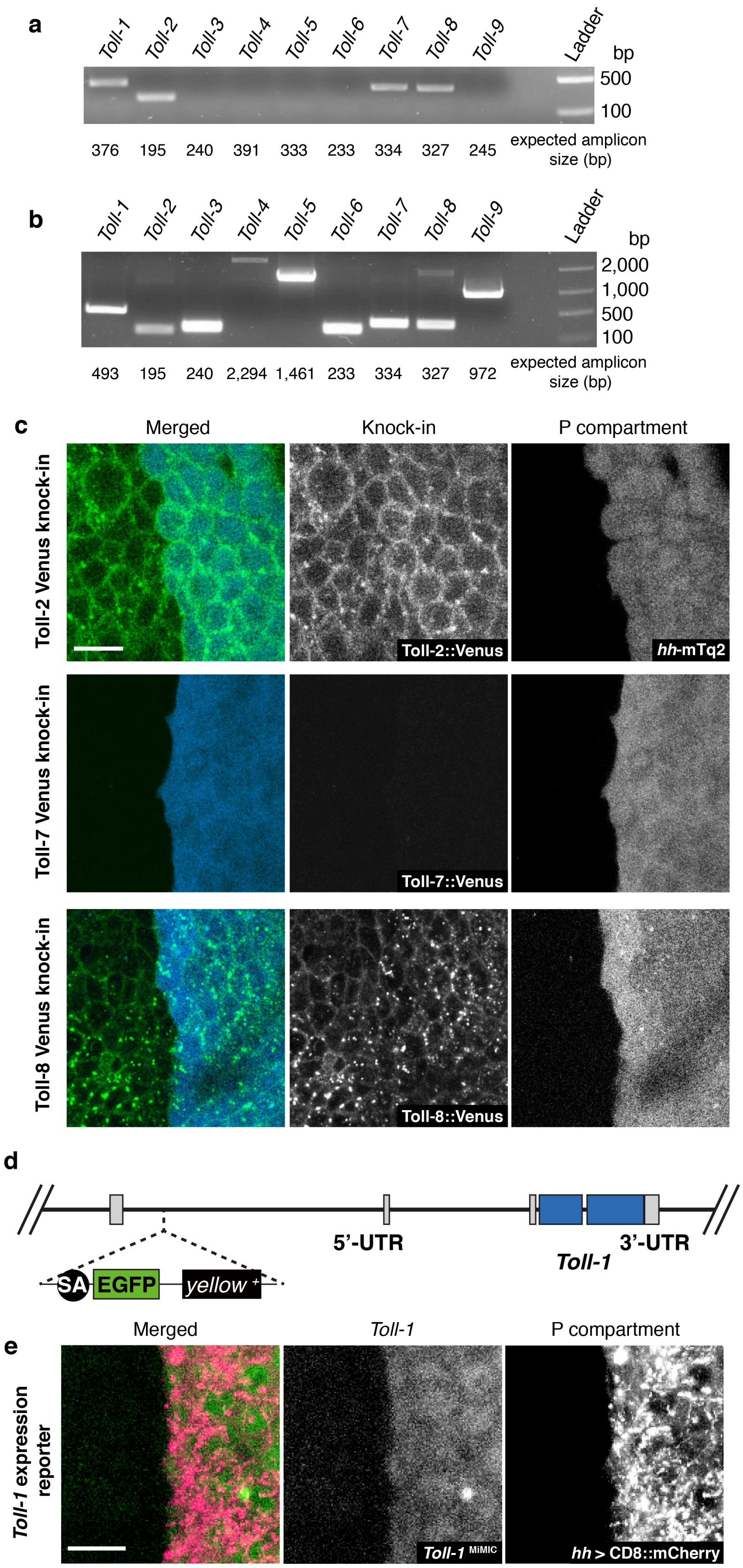

Toll family receptor genes other than Toll-1 do not display differential expression across the AP boundary.

a, RT-PCR for all Toll family receptor genes encoded in the *Drosophila* genome using total mRNA extracted from the abdominal histoblasts. Experiment was repeated two times with similar results. **b**, The primer sets used for RT-PCR in **a** were verified to work for PCR using genomic DNA as a template. Experiment was repeated two times with similar results. **c**, None of the Venus knock-in transgenic fly lines generated for *Toll-2*, *-7*, or *-8* (green) displayed a compartment-specific expression pattern. Posterior histoblasts were labeled with *hh*::mTq2 (cyan) to visualize the AP boundary. Presented data are representative images of n ≥ 3 animals for each gene. Scale bar: 10 μm. **d**, A map for the insertion point of $\mathit{Tl}^{\mathrm{MiMIC}}$ reporter line. Note that this reporter line would mimic the transcription of *Tl* but not the protein expression of its gene product since the reporter construction with a splicing acceptor sequence (SA) is inserted between the 1st and 2nd exons encoding for 5'-UTR of the *Tl* gene. **e**, Reporter expression for *Tl* using $\mathit{Toll\text{-}1}^{\mathrm{MiMIC}}$ (green). Posterior histoblasts were labeled with the membrane-anchored fluorescent protein mCherry (CD8::mCherry, magenta). Presented data is a representative image of n > 10 animals. Scale bar: 10 μm.

Supplementary Figure 2

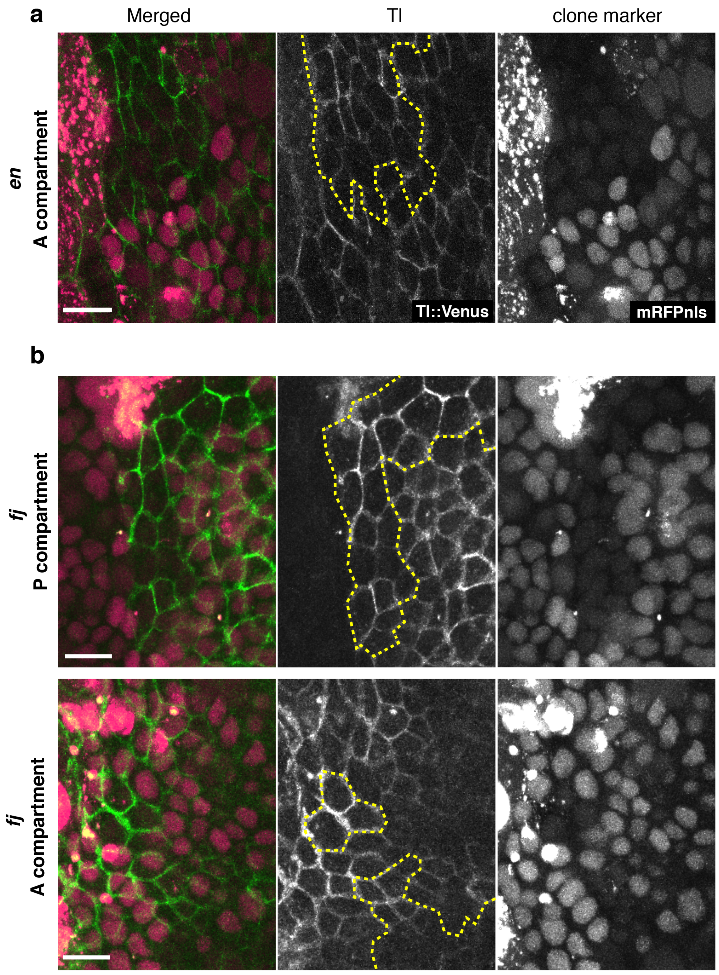

Regulation of Toll-1 expression in the A compartment is independent of *en*, and Toll-1 expression and membrane localization are not regulated by *four jointed*.

a, Mosaic analysis of Tl expression in *engrailed* (*en*) mutant cells in the A compartment. Tl expression was monitored with the Venus knock-in (green). Cells mutant for *en* were labeled with the loss of the marker expression, monomeric red fluorescent protein with nuclear localization signal (mRFPnls) (magenta). The anterior Tl expression is not affected in *en* clones. Presented data is a representative image of n ≥ 5 clones. Scale bar: 10 µm. **b**, Mosaic analysis of Tl expression in *four jointed* (*fj*) mutant cells in the P (top) and the A (bottom) compartments. The expression and localization of Tl::Venus were unaffected in *fj*

clones in the both compartments. Clones were outlined with yellow dashed lines. Presented data are representative images of $n \geq 5$ clones. Scale bars: 10 μm.

Supplementary Figure 3

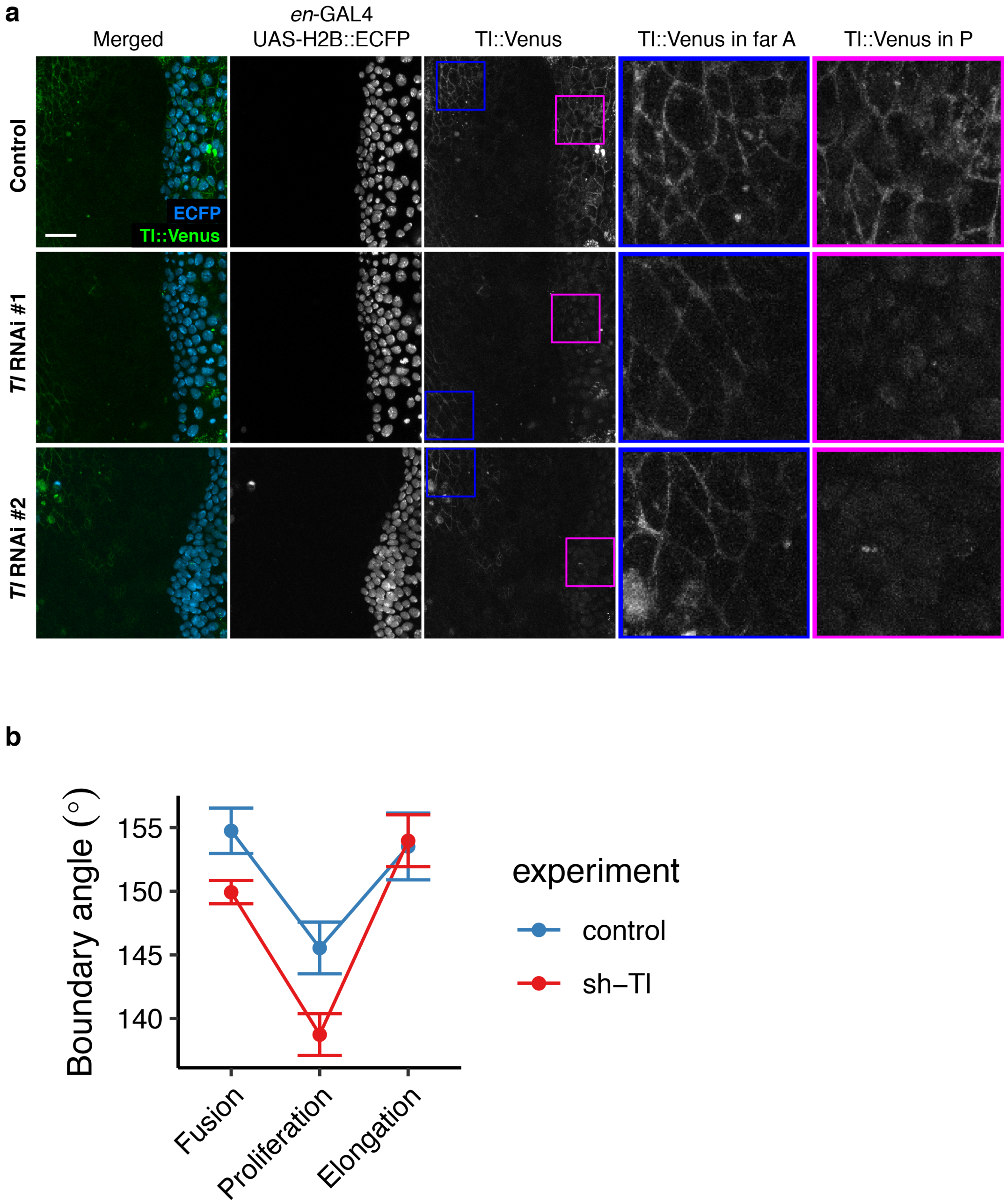

RNAi induced by short hairpin expression effectively knocks down *Toll-1*, and the mean boundary angle changes as the tissue moves through developmental stages. **a**, Short hairpin RNAs targeting the *Tl* gene sequence were genetically induced in histoblasts of the P compartment using an *en*-GAL4 driver. GAL4 expressing cells were marked by the expression of nuclear localized ECFP, and *Tl* expression was

monitored by *Tl*::Venus knock-in. In control tissue, posterior compartment *Tl* expression (top, magenta box) was comparable to the far anterior *Tl* expression in the A compartment (top, blue box). In the animals expressing short hairpin RNA targeting *Tl*, the posterior compartment expression of *Tl* (middle and bottom, magenta boxes) was severely reduced relative to the intact anterior expression of *Tl* (middle and bottom, blue boxes). Scale bar: 20 μm. **b**, The mean boundary angle at different developmental phases. The developmental phases were defined in Methods. The mean boundary angle was larger at fusion than at the proliferation phase both in control and *Tl* RNAi tissues. However, the *Tl* RNAi boundary was already less straight at the fusion. In the later phase (elongation phase), the boundary became more straight again, and no difference between control and *Tl* RNAi was observed, suggesting the presence of another mechanism involved in straightening the boundary at that stage. Control: n=6, *Tl* RNAi: n=8 boundaries. Data are presented as mean values +/- SEM.

Supplementary Figure 4

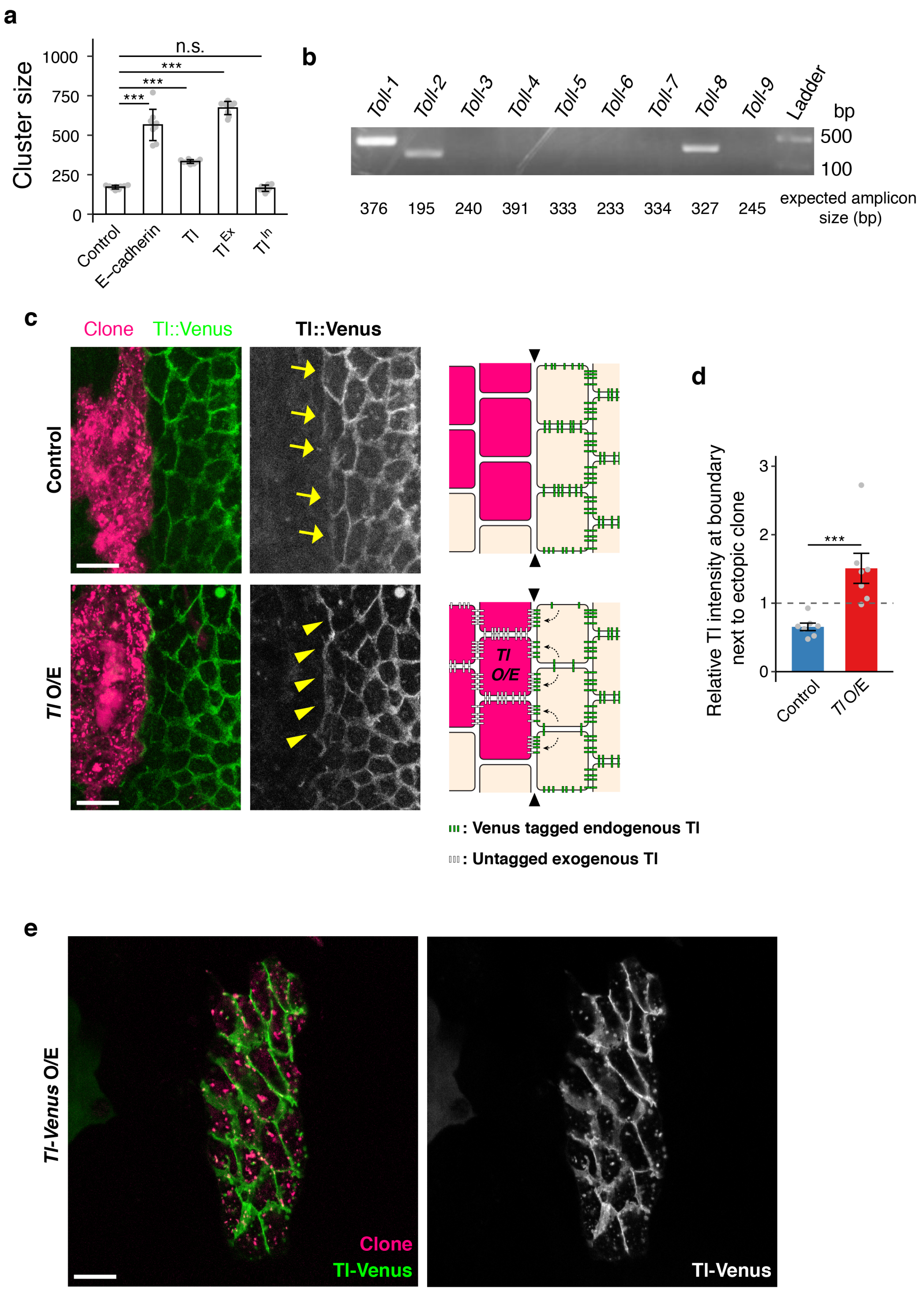

S2 cells express several Toll family receptor genes and *trans* interaction of Toll-1 mediates Toll-1 membrane localization.

a, Cluster size analysis for S2 cell aggregation assays presented in Fig. 3d. Average cluster size for S2 cells expressing Venus alone, *E-cadherin*, *Tl*, Tl^{Ex}, and Tl^{In} was

plotted. Data are presented as mean values +/- SD. The *p*-values were from a Mann-Whitney U-test (two-sided). ***: $p < 0.001$. n = 9 independent replicates for each experiment. **b**, RT-PCR using RNA isolated from S2 cells for all Toll family receptor genes encoded in the *Drosophila* genome. The expression of all *Drosophila* Toll family receptor genes in S2 cells were examined by RT-PCR using total RNA prepared from S2 cells. Expression of *Tl*, *Toll-2*, and *Toll-8* were detected. Experiment was repeated two times with similar results. **c**, Tl expressed in the neighboring compartment non cell-autonomously recruits Tl to the plasma membrane. Clones either expressing or not expressing Tl (magenta) were generated adjacent to the AP boundary in the A histoblasts. Endogenous Tl protein localization was monitored with the Tl Venus knock-in (green). Presented data is a representative image of n ≥ 3 clones. Scale bar: 10 μm. **d**, Relative signal intensity of Tl::Venus at the AP boundary adjacent to clones expressing either *Tl* or a clone marker alone. n = 8 cells for each experiment. Data are presented as mean values +/- SEM. The *p*-value was from a Mann-Whitney U-test (two-sided). ***: $p < 0.001$. **e**, Tl does not localize at the edges of the clone ectopically expressing Tl, tagged with the Venus fluorescent protein (Tl-Venus). The cells expressing Tl-Venus (green) were also labeled with the CD8::mCherry fluorescent protein (magenta). Presented data is a representative image of n ≥ 3 clones. Scale bar: 10 μm.

Supplementary Figure 5

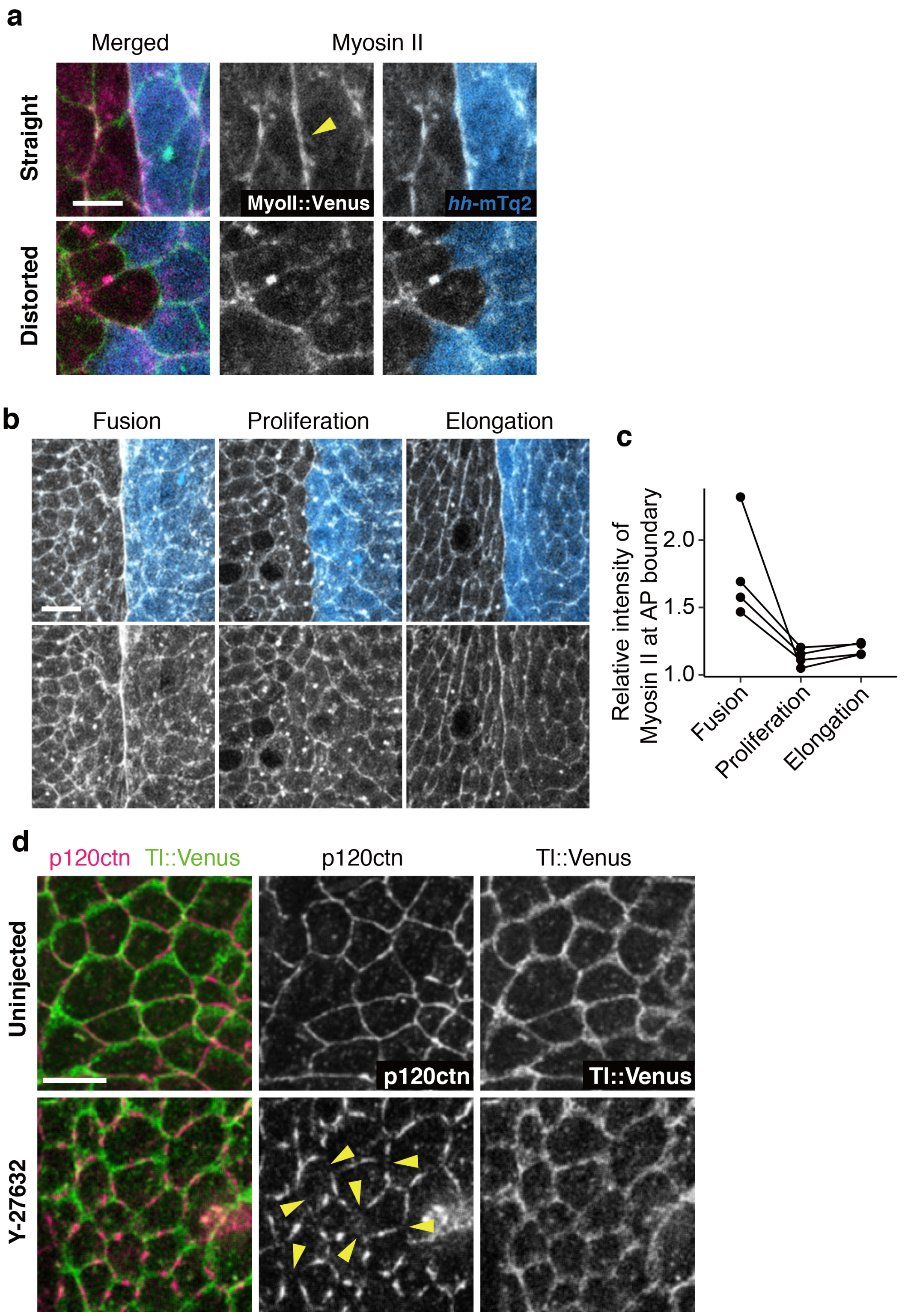

Toll-1 does not regulate enrichment of Myosin II on cell junctions along the AP boundary to straighten it.

a, Enrichment of Myosin II on cell junctions along the AP boundary when the boundary is straight and is distorted. Adherens junctions, Myosin II, and P histoblasts were visualized by p120ctn::tagRFP (green), Myosin II::Venus (magenta), and *hh*-mTurquoise2 (cyan), respectively. Yellow arrowheads indicate cable-like Myosin II accumulations. Presented data are representative snaphots of movies taken from ≥ 5 AP boundaries. Scale bar: 5 μm. **b**, Myosin II enrichment on cell junctions along the AP boundary over the course of abdominal histoblast morphogenesis. Scale bar: 10 μm. **c**, Signal intensity of Myosin II::Venus on junctions along the AP boundary relative to the average signal intensity on junctions one cell row away from the boundary on each side at each phase. **d**, Tl membrane localization is regulated by a distinct mechanism from that which regulates the localization of the adherens junction components. While the adherens junction labeled by p120ctn::tagRFP (magenta) became discontinuous upon application of the Rok inhibitor Y-27632, Tl::Venus (green) still localized at cell contacts. The slightly fuzzy Tl::Venus localization upon Y-27632 treatment may reflect changes in plasma membrane structure or membrane trafficking due to the altered contractility or mis-localization of AJ components. Experiment was repeated at least 3 times with similar results. Scale bar: 10 μm.

Supplementary Figure 6

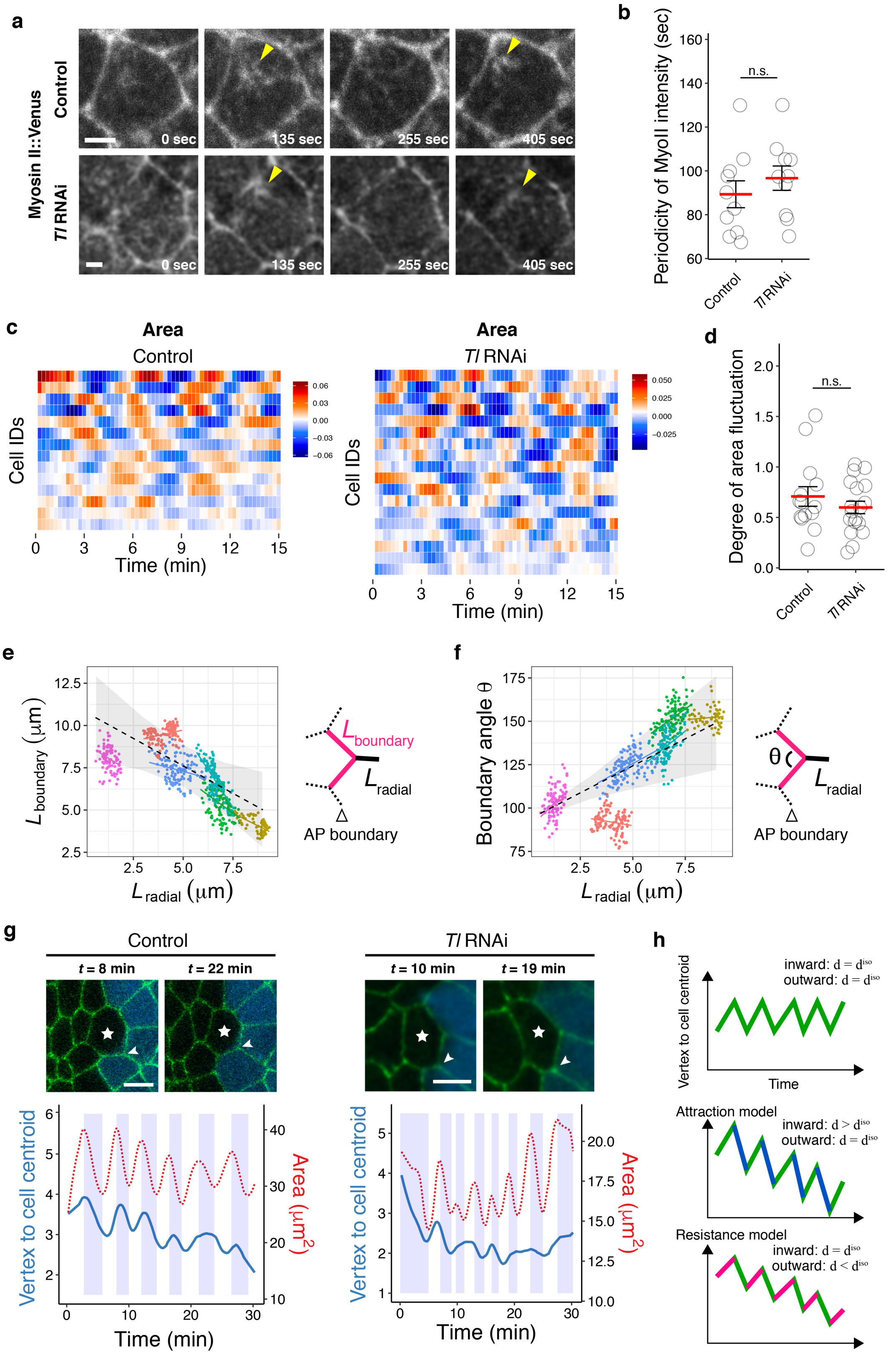

Apicomedial Myosin II population displays pulsed coalescence.

a, Myosin II labeled with MyoII::Venus exhibits both junctional and apicomedial localization within histoblasts. The apicomedial population coalesces periodically (yellow arrowhead) in both control and *Tl* RNAi cells (25 hAPF). Scale bar: 2 μm. **b**, Periodic coalescence of apicomedial Myosin II is intact in the *Tl* RNAi tissue. Periodicity of Myosin II signal intensity change in *Tl* RNAi cells is comparable to that of control. Data are presented as mean values +/- SEM. n = 10 cells for each experiment. The *p*-value was from a Mann-Whitney U-test (two-sided). n.s.: not significant. **c**, Dynamics of cell area for control and *Tl* RNAi tissues. The cell area profiles of A cells located at the AP boundary are shown for control (n=14 cells) and *Tl* RNAi (n=18 cells). Note that *Tl* RNAi cells also display area fluctuation. **d**, Degree of area fluctuation. The degree of area fluctuation of *Tl* RNAi cells is comparable to that of control. Data are presented as mean values +/- SEM. Control: n = 14 cells, *Tl* RNAi: n = 18 cells. The *p*-value was from a Mann-Whitney U-test (two-sided). n.s.: not significant. **e**, Correlation between a pair of junction length and its connected radial junction length. The total length of a pair of adjacent junctions on the AP boundary becomes shorter as their connected radial junction becomes longer, suggesting the junction length changes are caused by vertex sliding instead of independent elongation and contraction of three junctions connected at a vertex on the AP boundary. Each colored point indicates the relationship between an analyzed radial junction and its connected boundary junctions analyzed over the entire duration of observation. Dashed line is the fixed effect obtained from a mixed linear model showing an overall negative correlation. All radial junctions of three protruding cells during retraction were analyzed. The shaded area denotes the error band for 95% confidence intervals. Data presented for n = 6 boundary junction pairs. **f**, Correlation between the boundary angle of

adjacent boundary junctions and their connected radial junctions. The boundary angle of a pair of adjacent junctions on the AP boundary becomes larger as their connected radial junction becomes longer, suggesting that the elongation of radial junctions is accompanied by straightening of the AP boundary. Each colored point indicates the relationship between an analyzed radial junction and the boundary angle of its connected boundary junctions analyzed over an entire duration of observation. Dashed line is the fixed effect obtained from a mixed linear model showing an overall positive correlation. All radial junctions of three protruding cells during retraction were analyzed. The shaded area denotes the error band for 95% confidence intervals. Data presented for n = 6 boundary junction pairs. **g**, Dynamics of vertex motion at the AP boundary. Vertices at the AP boundary undergo pulsating radial displacement in control as well as in *Tl* RNAi tissues. Note that the vertex at the AP boundary moves toward the cell centroid as the cell (asterisk) at the AP boundary undergoes pulsatile contraction; the vertex motion toward the cell centroid was not evident in the *Tl* knock-down tissue. The distance from vertex to centroid was determined by tracking position of vertices relative to the cell centroid at the initial time point. Representative examples were shown for control (22 hAPF) and *Tl* knock-down animals (24 hAPF) in the proliferation phase. Scale bar: 5 μm. **h**, Two possible models that could explain the net centripetal displacement of vertices. Either inward sliding is more effective during contraction, or outward sliding is less effective during expansion.

Supplementary Table

All primers and oligo DNA used in this study are listed in this table.

Name	Sequence	Purpose
Toll-1 forward primer	5'-ACCACCAATCTGACGGATCT-3'	RT-PCR
Toll-1 reverse primer	5'-AGCCCAGCGAGCTAATGTT-3'	RT-PCR
Toll-2 forward primer	5'-GCCAACTAGCAAATCACCAAA-3'	RT-PCR
Toll-2 reverse primer	5'-GAGGTCAAGTGGCGTTCC-3'	RT-PCR
Toll-3 forward primer	5'-ATGACTTGGAATTAAACATAGACTGC-3'	RT-PCR
Toll-3 reverse primer	5'-TGAAGCTTACATCAAGATAGTCCAG-3'	RT-PCR
Toll-4 forward primer	5'-AATTGGGGTGGTGCAATAAC-3'	RT-PCR
Toll-4 reverse primer	5'-CGAATTCCAAGCACAGAAATTA-3'	RT-PCR
Toll-5 forward primer	5'-CAAAGGCCTGGGGAATCTA-3'	RT-PCR
Toll-5 reverse primer	5'-TCCACGATCAGACTACCTACACTC-3'	RT-PCR
Toll-6 forward primer	5'-CGCACAACAAGTTGACCAAA-3'	RT-PCR
Toll-6 reverse primer	5'-AATCAGCGCATTATTGTCCA-3'	RT-PCR
Toll-7 forward primer	5'-AGCTTCAGGAGGAGATTCGAG-3'	RT-PCR
Toll-7 reverse prime	5'-TGACGGGTGGTCAAGTTGT-3'	RT-PCR
Toll-8 forward primer	5'- CTGTTCGACGCCTTCGTT-3'	RT-PCR
Toll-8 reverse primer	5'-ATCGGGATCGAGTTCCTTCT-3'	RT-PCR
Toll-9 forward primer	5'- CGATCCAGGATGGCTTGATA-3'	RT-PCR
Toll-9 reverse primer	5'-ACCGTGGGATAGATCCAAATAC-3'	RT-PCR
Toll-1 gRNA F	5'-CTTCGCGACGTATAGGACTGGGAGA-3'	guide RNA for CRISPR/Cas9 knock-in
Toll-1 gRNA R	5'-AAACTCTCCCAGTCCTATACGTCGC-3'	guide RNA for CRISPR/Cas9 knock-in
Toll-2 gRNA F	5'-CTTCGATAGGGGGAGTTTTTAGACC-3'	guide RNA for CRISPR/Cas9 knock-in
Toll-2 gRNA R	5'-AAACGGTCTAAAAACTCCCCCTATC-3'	guide RNA for CRISPR/Cas9 knock-in
Toll-7 gRNA F	5'-CTTCGTATCTGGTGTAAAGAGGCG-3'	guide RNA for CRISPR/Cas9 knock-in
Toll-7 gRNA R	5'-AAACCGCCTCTTTACACCAGATAC-3'	guide RNA for CRISPR/Cas9 knock-in
Toll-8 gRNA F	5'-CTTCGAAATCTGCACATGTGAGTG-3'	guide RNA for CRISPR/Cas9 knock-in
Toll-8 gRNA R	5'-AAACCACTCACATGTGCAGATTTC-3'	guide RNA for CRISPR/Cas9 knock-in
sqh gRNA F	5’-CTTCGTTACTGCTCATCCTTGTCCT-3’	guide RNA for CRISPR/Cas9 knock-in
sqh gRNA R	5’-AAACAGGACAAGGATGAGCAGTAAC-3’	guide RNA for CRISPR/Cas9 knock-in
hh gRNA F	5’-CTTCGAACGAGTCTTAGATAAATCA-3’	guide RNA for CRISPR/Cas9 knock-in
hh gRNA R	5’-AAACTGATTTATCTAAGACTCGTTC-3’	guide RNA for CRISPR/Cas9 knock-in

Toll-1 HR arm Left F	5'-GCTTGATATCGAATTCGGCACTGAACGAAGGACGCTCG-3'	homologous arm for CRISPR/Cas9 knock-in
Toll-1 HR arm Left R	5'-AGTTGGGGGCGTAGGTACGTCGCTCTGTTTGGCATTCGTG-3'	homologous arm for CRISPR/Cas9 knock-in
Toll-1 HR arm Right F	5'-TAGTATAGGAACTTCGACTGGGAGAAGGCGGAGCTGTT-3'	homologous arm for CRISPR/Cas9 knock-in
Toll-1 HR arm Right R	5'-CGGGCTGCAGGAATTCGCAAGTGGTTCACCTAGTTGTAGGC-3'	homologous arm for CRISPR/Cas9 knock-in
Toll-2 HR arm Left F	5'-GCTTGATATCGAATTCGCACCGAGGAAATGGAGGCGAAC-3'	homologous arm for CRISPR/Cas9 knock-in
Toll-2 HR arm Left R	5'-AGTTGGGGGCGTAGGGACCAGGAAAGCTTGGCCGTTC-3'	homologous arm for CRISPR/Cas9 knock-in
Toll-2 HR arm Right F	5'-TAGTATAGGAACTTCAAACTCCCCCTATGGCCATATCC-3'	homologous arm for CRISPR/Cas9 knock-in
Toll-2 HR arm Right R	5'-CGGGCTGCAGGAATTCGGTTTACGTTCTTTACGCCTGGC-3'	homologous arm for CRISPR/Cas9 knock-in
Toll-7 HR arm Left F	5'-GCTTGATATCGAATTCGCGGATGCAGTTGGAATTGGTGC-3'	homologous arm for CRISPR/Cas9 knock-in
Toll-7 HR arm Left R	5'-AGTTGGGGGCGTAGGCACCAGATACGCCTGAACATGGG-3'	homologous arm for CRISPR/Cas9 knock-in
Toll-7 HR arm Right F	5'-TAGTATAGGAACTTCAGAGGCGTGGGCCAAAACCGAC-3'	homologous arm for CRISPR/Cas9 knock-in
Toll-7 HR arm Right R	5'-CGGGCTGCAGGAATTCCATGGAAACTCCATCTCAACTCGGG-3'	homologous arm for CRISPR/Cas9 knock-in
Toll-8 HR arm Left F	5'-GCTTGATATCGAATTCAGGAACTCGATCCCGATCTTCGC-3'	homologous arm for CRISPR/Cas9 knock-in
Toll-8 HR arm Left R	5'-AGTTGGGGGCGTAGGCATGTGCAGATTTCTAGACGCCGG-3'	homologous arm for CRISPR/Cas9 knock-in
Toll-8 HR arm Right F	5'-TAGTATAGGAACTTCGTGGGGTTAGCTTTAAGCAGAGGG-3'	homologous arm for CRISPR/Cas9 knock-in
Toll-8 HR arm Right R	5'-CGGGCTGCAGGAATTCGCTGCCTGTTCTTTCCCCATTCG-3'	homologous arm for CRISPR/Cas9 knock-in
sqh HR arm Left F	5'-GCTTGATATCGAATTCATCAGGCGCAGATTGCC-3'	homologous arm for CRISPR/Cas9 knock-in
sqh HR arm Left R	5'-AGTTGGGGGCGTAGGCTGCTCATCCTTGTCCTTGGC-3'	homologous arm for CRISPR/Cas9 knock-in
sqh HR arm Right F	5'-TAGTATAGGAACTTCATCGCCAGCAGTCGATTCACTAGC-3'	homologous arm for CRISPR/Cas9 knock-in
sqh HR arm Right R	5'-CGGGCTGCAGGAATTCATCACATCGTGCTAGTGATCATGC-3'	homologous arm for CRISPR/Cas9 knock-in
hh HR arm Left F	5'-GCTTGATATCGAATTCCACAACGCGGAATGAACTCGAGG-3'	homologous arm for CRISPR/Cas9 knock-in
hh HR arm Left R	5'-AGTTGGGGGCGTAGGCATGATTTATCTAAGACTCGTTGTTTGC-3'	homologous arm for CRISPR/Cas9 knock-in
hh HR arm Left F	5'-TAGTATAGGAACTTCGATAACCACAGCTCAGTGCCTTGG-3'	homologous arm for CRISPR/Cas9 knock-in
hh HR arm Left R	5'-CGGGCTGCAGGAATTCCCTTGCTCATCAAGCGATCCGC-3'	homologous arm for CRISPR/Cas9 knock-in
Toll-1FL F	5'- GGCCGCGGCTCGAGGGTACCAACTTAAAAAAAAAAATCAAAATGAGTCGACTAAAGGCCGCTTC -3'	UAS constract
Toll-1FL R	5'- AACGATTCATTCTAGACTATACGTCGCTCTGTTTGGC -3'	UAS constract
Toll-1Int F	5'- GGCCGCGGCTCGAGGGTACCAACTTAAAAAAAAAAATCAAAATGGAAAAGGGCGTGTTCATAGC -3'	UAS constract
Toll-1Int R	5'- AACGATTCATTCTAGACTATACGTCGCTCTGTTTGGC -3'	UAS constract
Toll-1Ex F	5'- GGCCGCGGCTCGAGGGTACCAACTTAAAAAAAAAAATCAAAATGAGTCGACTAAAGGCCGCTTC -3'	UAS constract
Toll-1Ex R	5'- AACGATTCATTCTAGATTACGTCTGGAATTTGTAGTACAGTG -3'	UAS constract
Toll-1FL F	5'- GGCCGCGGCTCGAGGGTACCAACTTAAAAAAAAAAATCAAAATGAGTCGACTAAAGGCCGCTTC -3'	UAS constract with Venus tag
Toll-1FL R	5'- GCCCTTGCTCACCATTACGTCGCTCTGTTTGGC-3'	UAS constract with Venus tag
Toll-1Int F	5'- GGCCGCGGCTCGAGGGTACCAACTTAAAAAAAAAAATCAAAATGGAAAAGGGCGTGTTCATAGC -3'	UAS constract with Venus tag
Toll-1Int R	5'- GCCCTTGCTCACCATTACGTCGCTCTGTTTGGC-3'	UAS constract with Venus tag

Toll-1Ex F	5'- GGCCGCGGCTCGAGGGTACCAACTTAAAAAAAAAAAATCAAAATGAGTCGACTAAAGGCCGCTTC -3'	UAS constract with Venus tag
Toll-1Ex R	5'- GCCCTTGCTCACCATCGTCTGGAATTTGTAGTACAGTG-3'	UAS constract with Venus tag
Venus F	5'- ATGGTGAGCAAGGGCGAG-3'	UAS constract with Venus tag
Venus R	5'- AACGATTCATTCTAGATTACTTGTACAGCTCGTCCATG -3'	UAS constract with Venus tag
Mir6.1-Tl-F	5’-GGCAGCTTACTTAAACTTAATCACAGCCTTTAATGTGCAGGTGGTTCCAACTTATCTCTAAGTTAATATACCATATC-3’	shRNA construct
Mir6.1-Tl-R	5'- AATAATGATGTTAGGCACTTTAGGTACGCAGGTGGTTCCAACTTATATCTAGATATGGTATATTAACTTAGAGA-3'	shRNA construct
