# Supplementary material for: Differential cell adhesion implemented by Drosophila Toll corrects local distortions of the anterior-posterior compartment boundary
Source: Nat Commun. 2020 Dec 10;11:6320. doi: 10.1038/s41467-020-20118-y (PMC7729853; doi:10.1038/s41467-020-20118-y)
Supplement: Supplementary file 2 — Description of Additional Supplementary Files [file 41467_2020_20118_MOESM2_ESM.pdf]

Description of Additional Supplementary Files

File Name: Supplementary Movie 1
Description: Pulsed coalescence of medial Myosin II in control histoblasts.

File Name: Supplementary Movie 2
Description: Pulsed coalescence of medial Myosin II in Toll-1 knockdown histoblasts.

File Name: Supplementary Movie 3
Description: Pulsed contraction of an A cell at the distorted AP boundary in a control animal.

File Name: Supplementary Movie 4
Description: Pulsed contraction of an A cell at the distorted AP boundary in a Toll-1 knockdown animal.
