# Supplementary material for: Differential cell adhesion implemented by Drosophila Toll corrects local distortions of the anterior-posterior compartment boundary
Source: Nat Commun. 2020 Dec 10;11:6320. doi: 10.1038/s41467-020-20118-y (PMC7729853; doi:10.1038/s41467-020-20118-y)
Supplement: Supplementary file 7 — Reporting Summary [file 41467_2020_20118_MOESM7_ESM.pdf]

nature research

Corresponding author(s): Daiki Umetsu and Erina Kuranaga

Last updated by author(s): Oct 23, 2020

Reporting Summary

Nature Research wishes to improve the reproducibility of the work that we publish. This form provides structure for consistency and transparency in reporting. For further information on Nature Research policies, see our Editorial Policies and the Editorial Policy Checklist.

Statistics

For all statistical analyses, confirm that the following items are present in the figure legend, table legend, main text, or Methods section.

n/a	Confirmed	
☐	☒	The exact sample size (n) for each experimental group/condition, given as a discrete number and unit of measurement
☒	☐	A statement on whether measurements were taken from distinct samples or whether the same sample was measured repeatedly
☐	☒	The statistical test(s) used AND whether they are one- or two-sided<br>*Only common tests should be described solely by name; describe more complex techniques in the Methods section.*
☒	☐	A description of all covariates tested
☒	☐	A description of any assumptions or corrections, such as tests of normality and adjustment for multiple comparisons
☐	☒	A full description of the statistical parameters including central tendency (e.g. means) or other basic estimates (e.g. regression coefficient) AND variation (e.g. standard deviation) or associated estimates of uncertainty (e.g. confidence intervals)
☐	☒	For null hypothesis testing, the test statistic (e.g. F, t, r) with confidence intervals, effect sizes, degrees of freedom and P value noted<br>*Give P values as exact values whenever suitable.*
☒	☐	For Bayesian analysis, information on the choice of priors and Markov chain Monte Carlo settings
☒	☐	For hierarchical and complex designs, identification of the appropriate level for tests and full reporting of outcomes
☒	☐	Estimates of effect sizes (e.g. Cohen's d, Pearson's r), indicating how they were calculated

Our web collection on statistics for biologists contains articles on many of the points above.

Software and code

Policy information about availability of computer code

Data collection	This study uses an image processing plug-in, Tissue Analyzer, of the open source image processing software ImageJ (version 2.0.0) for the automation of cell shape detection and quantitative data production. Automated detections of cell shape were performed by applying a common parameter set for all cells within each image to avoid psychological biases.
Data analysis	This study uses custom codes generated in R (4.0.0) for the data analysis and plotting. However, the codes themselves do not include experimental results and are used only for help automate batch processes and visualization of graphs.

For manuscripts utilizing custom algorithms or software that are central to the research but not yet described in published literature, software must be made available to editors and reviewers. We strongly encourage code deposition in a community repository (e.g. GitHub). See the Nature Research guidelines for submitting code & software for further information.

Data

Policy information about availability of data

All manuscripts must include a data availability statement. This statement should provide the following information, where applicable:

- Accession codes, unique identifiers, or web links for publicly available datasets
- A list of figures that have associated raw data
- A description of any restrictions on data availability

The data that support all experimental findings of this study are available within the paper and its Supplementary Information files or from the corresponding author D.U. upon request. Source data are provided with this paper. Raw data necessary to reproduce all statistical analyses and results in the paper as well as P values for all figures are provided in the source data file.

Field-specific reporting

Please select the one below that is the best fit for your research. If you are not sure, read the appropriate sections before making your selection.

- [x] Life sciences
- [ ] Behavioural & social sciences
- [ ] Ecological, evolutionary & environmental sciences

For a reference copy of the document with all sections, see nature.com/documents/nr-reporting-summary-flat.pdf

Life sciences study design

All studies must disclose on these points even when the disclosure is negative.

Sample size	The sample sizes were not predetermined by any statistical methods. Since most of the analysis performed in this study involve image processing of large datasets, the sample sizes were maximized under the limitation by the time and manpower resources required for image analysis.
Data exclusions	No data were excluded from the analysis.
Replication	All images were obtained from multiple animals ($n \geq 3$). Experiments with different genotypes were repeated and confirmed in multiple animals ($n \geq 3$). The experiments were performed at least two times. The observed phenotypes were highly reproducible as indicated by the significance of p-values obtained by statistical tests.
Randomization	The samples were not randomized since the experiments were either categorized by quantitative values obtained by objective and systematic image processing methods using plugins of the ImageJ software or by genotypes.
Blinding	The investigators were not blinded during data collection or analysis. This is not required given that the data were collected from all cells or cell junctions within the entire region of each image.

Reporting for specific materials, systems and methods

We require information from authors about some types of materials, experimental systems and methods used in many studies. Here, indicate whether each material, system or method listed is relevant to your study. If you are not sure if a list item applies to your research, read the appropriate section before selecting a response.

Materials & experimental systems

n/a	Involved in the study	
x		Antibodies
	x	Eukaryotic cell lines
x		Palaeontology and archaeology
	x	Animals and other organisms
x		Human research participants
x		Clinical data
x		Dual use research of concern

Methods

n/a	Involved in the study	
x		ChIP-seq
x		Flow cytometry
x		MRI-based neuroimaging

Eukaryotic cell lines

Policy information about cell lines

Cell line source(s)	Drosophila S2 cells
Authentication	The Drosophila S2 cell line used in this study was not authenticated.
Mycoplasma contamination	The cell line was not tested for mycoplasma contamination.
Commonly misidentified lines (See ICLAC register)	N/A

Animals and other organisms

Policy information about studies involving animals; ARRIVE guidelines recommended for reporting animal research

Laboratory animals	Drosophila melanogaster was used in this study. Both males and females were used for analyses.
Wild animals	N/A

Field-collected samples	N/A
Ethics oversight	N/A

Note that full information on the approval of the study protocol must also be provided in the manuscript.
